# Supplementary material for: Metabolomic Analyses to Identify Candidate Biomarkers of Cystinosis
Source: Int J Mol Sci. 2023 Jan 30;24(3):2603. doi: 10.3390/ijms24032603 (PMC9916752; doi:10.3390/ijms24032603)
Supplement: Supplementary file 1 [file ijms-24-02603-s001.zip › Supplementary File S1-Results-.pdf]

S1. Data analysis of GC-MS, LC-qTOF-MS and LC-MS/MS based metabolomics analyzes

S1.1. Plasma Samples

The data matrices were transferred to an Excel work file and normalized to total peak area in order to eliminate day to day variations from multiple analytical batches. Missing values in the data table were filled with the half value of the smallest concentration in the metabolite group. After transferring this data matrix to the SIMCA-P + (ver. 13.0) program, multivariate statistical analysis was performed by using principal component analysis (PCA) (unsupervised) to monitor any outlier or any systematic error in the data and partial least squares discriminant analysis (PLS-DA) (supervised) methods to investigate the metabolomics profile differences between the groups in detail. A variable importance in projection (VIP) graph was also utilized in order to identify the metabolites affecting differentiation in the PLS-DA score plot, and the coefficients obtained from regression analysis were evaluated to determine the relative changes of metabolite levels with treatment. Furthermore, in order to detect the metabolites that differ between groups, first of all, the Shapiro-Wilk test was used to assess whether the metabolites had a normal distribution. The t-test was utilized if the two groups being compared were normally distributed; otherwise, the Mann-Whitney U non-parametric test was used **(Table S1.1-2)**. Finally, using the metabolites acquired, pathway analyzes were performed to determine which pathway changed the most with the phenotype.

Table S1.1. Statistical analysis results of metabolites identified by GC-MS and LC-qTOF-MS based metabolomics analysis from plasma samples.

Metabolite	Method	Patient Group		Treatment Group			Healthy Group		
		Mean	Median	Mean (SD) or Median (IQR)	p value	Test*	Mean (SD) or Median (IQR)	p value	Test*
(2-hydroxyethoxy)sulfonic acid	LC-MS/MS(+)	1.007(0.057)	1.004(0.072)	1.006(0.040)	0.979	T	1.008(0.044)	0.934	T
(3beta,22E)-26,27-Dinorergosta-5,22-dien-3-ol	LC-MS/MS(+)	0.979(0.615)	0.866(0.793)	0.970(0.510)	0.966	T	0.787(0.492)	0.320	T
(S)-Oleuropeic acid	LC-MS/MS(+)	1.011(0.675)	1.195(1.287)	0.514(1.263)	0.739	M	1.145(1.144)	0.989	M
(Z)-1,3-Octadiene	LC-MS/MS(+)	0.702(0.853)	0.000(1.573)	0.000(1.362)	0.391	M	0.000(0.313)	0.320	M
1-(2-hydroxy-4,6-dimethoxyphenyl)ethanone	LC-MS/MS(+)	0.479(0.945)	0.000(0.435)	0.000(0.244)	0.867	M	0.000(0.000)	0.175	M
1-(9Z-octadecenoyl)-2-(5Z,8Z,11Z,14Z-eicosatetraenoyl)-3-O-alpha-D-glucuronyl-sn-glycerol	LC-MS/MS(+)	1.205(1.619)	0.678(1.725)	0.516(1.311)	0.584	M	0.623(1.317)	0.631	M
1,18-Diamino-5,9,14-triazaoctadecane	LC-MS/MS(+)	0.967(0.374)	0.885(0.451)	0.953(0.313)	0.710	M	0.949(0.333)	0.878	T
1,3-dihydroxyacetone	GC-MS	0.957(0.091)	0.956(0.063)	1.028(0.075)	0.037	T	1.009(0.099)	0.126	T
10-Hydroxydecanoate	LC-MS (-)	1.016(0.752)	0.576(0.580)	0.576(0.675)	0.943	M	0.576(0.852)	1.000	M
11'-Carboxy-gamma-chromanol	LC-MS/MS (-)	1.030(0.840)	0.740(0.982)	0.378(0.722)	0.309	M	0.378(1.227)	0.604	M
12-deoxyphorbol-13-acetate (prostratin)	LC-MS/MS(+)	0.409(1.325)	0.038(0.076)	0.005(0.060)	0.231	M	0.016(0.035)	0.169	M
12-HETE-G	LC-MS/MS (-)	0.719(0.534)	0.564(0.774)	0.718(0.847)	0.553	M	0.785(0.873)	0.571	M
16-Hydroxyhexadecanoic acid	LC-MS (-)	1.125(0.450)	1.069(0.605)	1.056(0.393)	0.657	T	1.102(0.437)	0.878	T
1alpha,25-dihydroxy-2beta-(3-hydroxypropoxy)vitamin D3	LC-MS/MS (-)	0.803(0.450)	0.586(0.000)	0.586(0.000)	0.053	M	0.878(1.450)	0.046	M
1-hexadecanol	GC-MS	0.879(0.348)	0.795(0.439)	0.803(0.659)	0.494	M	1.033(0.346)	0.172	M
1-O-Hexadecyl-lyso-sn-glycero-3-phosphocholine	LC-MS/MS(+)	0.717(1.104)	0.204(0.536)	0.436(0.279)	0.336	M	0.339(0.238)	0.321	M
2-(14,15-Epoxyeicosatrienoyl) Glycerol	LC-MS/MS(+)	0.930(0.529)	0.821(0.091)	0.783(0.117)	0.681	M	0.823(0.190)	0.864	M
2,3-Dihydro-5-methyl-3-thiophenethiol	LC-MS/MS (-)	1.063(0.168)	1.077(0.260)	1.018(0.129)	0.297	M	1.099(0.132)	0.782	M
2,4-Octadiene	LC-MS/MS(+)	0.872(0.495)	0.778(0.453)	0.794(0.371)	0.830	M	0.745(0.481)	0.927	M
20-Carboxy-leukotriene B4	LC-MS/MS(+)	0.514(0.822)	0.000(0.938)	0.447(0.638)	0.587	M	0.000(0.490)	1.000	M
20-oxo-heneicosanoic acid	LC-MS/MS(+)	0.890(1.060)	0.473(0.269)	0.445(0.114)	0.316	M	0.510(0.148)	0.927	M

Metabolite	Method	Patient Group		Treatment Group			Healthy Group		
		Mean	Median	Mean (SD) or Median (IQR)	p value	Test*	Mean (SD) or Median (IQR)	p value	Test*
2-amino-1-phenylethanol	GC-MS	0.711(0.252)	0.629(0.296)	1.040(0.616)	0.083	T	1.072(0.643)	0.023	T
2-Hydroxy-4-oxo-5,12-heneicosadien-1-yl acetate	LC-MS/MS(+)	0.957(0.272)	0.971(0.273)	1.049(0.326)	0.397	T	0.872(0.502)	0.702	M
2-hydroxybutyric acid	GC-MS	0.715(0.448)	0.527(0.455)	0.824(0.427)	0.169	M	1.240(0.616)	0.018	M
2-hydroxyphenylacetic acid	GC-MS	1.136(1.943)	0.365(0.711)	0.383(0.809)	0.981	M	0.195(0.143)	0.006	M
2-isopropylmalic acid	GC-MS	0.810(0.297)	0.742(0.212)	1.090(0.416)	0.085	M	0.827(0.326)	0.872	T
2-ketobutyric acid	GC-MS	1.088(1.117)	0.522(1.293)	0.583(0.695)	0.830	M	0.580(0.856)	0.948	M
2-ketoisocaproic acid	GC-MS	0.643(0.238)	0.594(0.295)	0.989(0.425)	0.016	T	1.035(0.676)	0.013	M
2-keto-L-gulonic acid	GC-MS	1.134(0.765)	0.849(0.693)	0.802(0.316)	0.793	M	0.675(0.756)	0.344	M
2-Octenoylcarnitine	LC-MS/MS(+)	0.983(1.187)	0.443(1.448)	0.361(0.944)	0.710	M	0.355(0.633)	0.864	M
2-Oxo-4-methylthiobutanoic acid	LC-MS/MS(+)	0.991(0.088)	0.989(0.087)	1.000(0.126)	0.421	M	0.999(0.122)	0.815	T
3,4-Dimethyl-5-pentyl-2-furanpropanoic acid	LC-MS/MS(+)	0.879(0.202)	0.830(0.232)	0.861(0.282)	0.200	M	0.897(0.259)	0.249	M
3-Aminoisobutanoic acid	LC-MS(+)	0.723(0.151)	0.674(0.166)	0.784(0.198)	0.246	M	0.761(0.190)	0.071	M
3-aminoisobutyric acid	GC-MS	1.236(1.383)	0.920(0.597)	1.221(0.999)	0.259	M	0.349(0.179)	0.000	M
3beta,7alpha-Dihydroxy-5-cholestenoate	LC-MS/MS (-)	0.767(0.846)	0.159(1.160)	0.726(1.542)	0.288	M	0.919(1.431)	0.263	M
3-Dehydroteasterone	LC-MS/MS (-)	1.115(1.097)	0.450(0.712)	0.450(0.559)	0.774	M	0.450(1.082)	0.845	M
3-Hydroxybutyric acid	LC-MS (-)	1.028(0.739)	0.916(0.736)	0.733(0.393)	0.676	M	1.270(0.734)	0.325	T
3-Hydroxyglutaric acid	LC-MS (-)	1.541(2.730)	0.738(0.234)	0.752(0.244)	0.766	M	0.748(0.373)	0.370	M
3-hydroxypropanoic acid	GC-MS	1.338(1.961)	0.528(0.474)	0.479(0.418)	0.616	M	0.391(0.256)	0.226	M
3-indolelactic acid	GC-MS	1.824(0.917)	2.008(1.209)	1.050(0.349)	0.012	T	0.351(0.271)	0.000	M
3-Indoxyl sulfate	LC-MS/MS (-)	1.721(2.749)	0.442(1.723)	0.402(0.322)	0.843	M	0.034(0.000)	0.000	M
3-methyl-2-oxobutanoic acid	GC-MS	0.895(0.374)	0.846(0.467)	0.900(0.538)	0.458	M	0.616(0.636)	0.267	M
3-methyl-L-histidine	GC-MS	1.040(0.276)	0.985(0.211)	1.021(0.264)	0.860	T	0.934(0.319)	0.820	M
3-phenyllactic acid	GC-MS	1.085(0.081)	1.065(0.072)	0.967(0.111)	0.002	M	0.997(0.098)	0.000	M
3-phosphoglycerate	GC-MS	0.301(0.211)	0.390(0.420)	0.259(0.586)	0.867	M	0.453(0.564)	0.253	M
4-hydroxy-3-methoxymandelic acid	GC-MS	1.113(0.763)	0.872(0.350)	0.965(0.348)	0.616	M	0.886(0.297)	0.603	M

Metabolite	Method	Patient Group		Treatment Group			Healthy Group		
		Mean	Median	Mean (SD) or Median (IQR)	p value	Test*	Mean (SD) or Median (IQR)	p value	Test*
4-hydroxy-L-proline	GC-MS	0.907(0.430)	0.911(0.625)	0.946(0.577)	0.458	M	0.730(0.363)	0.223	T
4-hydroxyphenylacetic acid	GC-MS	0.683(0.269)	0.720(0.359)	0.818(0.964)	0.325	M	0.781(0.755)	0.649	M
4-Hydroxyproline	LC-MS(+)	0.949(0.164)	0.940(0.235)	0.994(0.189)	0.279	M	1.005(0.313)	0.113	M
4-isopropylbenzoic acid	GC-MS	1.115(0.145)	1.058(0.232)	1.028(0.167)	0.162	T	0.914(0.174)	0.001	T
4-Trimethylammoniobutanoic acid	LC-MS(+)	0.777(0.362)	0.701(0.422)	0.670(0.416)	0.681	M	0.402(0.140)	0.000	M
5,8,11,14-Octadecatetraynoic acid	LC-MS/MS (-)	1.899(1.941)	1.049(2.054)	0.755(0.533)	0.336	M	0.305(0.202)	0.000	M
5-aminovaleric acid	GC-MS	0.013(0.004)	0.013(0.005)	1.215(3.285)	0.000	M	0.014(0.006)	0.302	T
5-hydroxy-L-tryptophan	GC-MS	0.740(0.276)	0.727(0.377)	1.044(1.266)	0.085	M	0.805(0.439)	0.580	M
5-Hydroxymethyluridine	LC-MS (-)	0.991(0.069)	0.988(0.077)	1.001(0.139)	0.793	T	1.044(0.152)	0.096	M
6-deoxy-D-glucose	GC-MS	1.141(0.586)	0.886(0.316)	0.884(0.400)	0.350	M	0.793(0.462)	0.179	M
6-phosphogluconic acid	GC-MS	0.953(0.081)	0.932(0.115)	1.072(0.168)	0.029	T	0.966(0.132)	0.312	M
7b-Hydroxy-3-oxo-5b-cholan-24-oate	LC-MS/MS (-)	0.595(0.547)	0.311(0.674)	0.875(1.103)	0.152	M	0.953(1.571)	0.079	M
8,11,14-Eicosatrienoic acid	LC-MS(+)	0.833(0.740)	0.673(0.567)	1.113(1.792)	0.356	M	0.528(0.798)	0.304	M
9-Decenoylcarnitine	LC-MS/MS(+)	0.688(0.624)	0.514(0.630)	0.758(0.422)	0.230	M	0.829(0.802)	0.228	M
9Z,12Z-Linoleic acid	LC-MS/MS (-)	1.093(3.079)	0.185(0.357)	0.032(0.226)	0.471	M	0.007(0.416)	0.458	M
Acetol	GC-MS	0.966(0.849)	0.636(0.853)	0.600(0.455)	0.943	M	0.462(0.359)	0.115	M
Acetyl-N-formyl-5-methoxykynurenamine	LC-MS/MS(+)	1.270(2.521)	0.219(0.778)	0.308(0.452)	0.275	M	0.014(0.053)	0.001	M
Aconitic acid	LC-MS (-)	1.001(0.456)	0.965(0.451)	0.994(0.163)	0.538	M	0.943(0.736)	0.904	M
Allo-inositol	GC-MS	0.677(1.719)	0.113(0.030)	0.151(0.648)	0.239	M	0.108(0.047)	0.312	M
Allolithocholic acid	LC-MS/MS (-)	1.167(1.249)	0.357(1.310)	0.357(1.045)	0.635	M	0.357(1.375)	0.892	M
Allose	GC-MS	0.989(0.528)	0.822(0.199)	0.857(0.322)	0.793	M	0.815(0.246)	0.922	M
Alloxan	LC-MS/MS(+)	0.994(0.043)	1.000(0.030)	1.009(0.042)	0.333	T	1.009(0.045)	0.297	T
Alpha ketoglutaric acid	GC-MS	0.830(0.277)	0.804(0.317)	1.025(0.318)	0.102	T	0.796(0.445)	0.673	M
Alpha-N-Phenylacetyl-L-glutamine	LC-MS(+)	0.858(0.712)	0.715(0.672)	0.796(1.067)	0.799	M	0.910(0.934)	0.664	M
Androsterone	GC-MS	0.692(0.285)	0.607(0.374)	1.157(0.536)	0.010	T	0.994(0.458)	0.020	T

Metabolite	Method	Patient Group		Treatment Group			Healthy Group		
		Mean	Median	Mean (SD) or Median (IQR)	p value	Test*	Mean (SD) or Median (IQR)	p value	Test*
Arachidic acid	GC-MS	0.882(0.090)	0.892(0.055)	0.999(0.151)	0.008	M	0.996(0.180)	0.000	M
Arachidonic acid	LC-MS/MS (-)	0.933(0.530)	0.744(0.000)	0.744(0.000)	0.865	M	0.744(0.000)	0.599	M
Arbutin	GC-MS	0.100(0.137)	0.050(0.068)	0.183(0.149)	0.043	M	0.054(0.081)	0.922	M
Aspartic acid	GC-MS	0.945(0.324)	0.913(0.453)	0.995(0.447)	0.741	T	1.083(0.507)	0.327	T
Aspartyl-Glutamate	LC-MS/MS(+)	0.072(0.130)	0.000(0.084)	0.000(0.247)	0.488	M	0.133(0.491)	0.037	M
Azelaic acid	LC-MS/MS (-)	1.141(0.907)	1.253(1.166)	0.672(1.334)	1.000	M	0.336(0.827)	0.049	M
Beta-alanine	GC-MS	1.068(0.777)	0.699(0.275)	0.767(0.707)	0.685	M	0.538(0.326)	0.006	M
Canavaninosuccinate	LC-MS/MS(+)	1.039(0.099)	1.052(0.046)	1.052(0.186)	0.799	M	1.038(0.128)	0.983	T
Capric acid	GC-MS	1.261(0.864)	1.125(1.277)	0.584(0.408)	0.068	M	1.123(0.396)	0.595	T
Caprylic acid	GC-MS	0.945(0.297)	0.831(0.255)	0.980(0.240)	0.740	T	0.940(0.252)	0.956	T
CE(24:0)	LC-MS/MS(+)	1.420(2.558)	0.250(1.640)	1.090(2.015)	0.631	M	0.114(0.798)	0.293	M
Cellobiose	GC-MS	0.952(1.177)	0.440(1.561)	0.194(0.137)	0.259	M	0.162(0.226)	0.093	M
Cholesta-4,6-dien-3-one	LC-MS(+)	0.932(0.431)	0.788(0.497)	1.089(0.832)	0.053	M	0.886(0.602)	0.644	M
Cholesterol	GC-MS	1.031(0.890)	0.791(1.180)	1.197(0.824)	0.621	T	0.765(1.408)	0.845	M
cis-Aconitic acid	LC-MS/MS(+)	0.992(0.042)	0.979(0.040)	1.002(0.040)	0.488	T	0.999(0.049)	0.610	T
Citraconic acid	LC-MS (-)	1.119(0.319)	1.038(0.196)	1.076(0.184)	0.681	M	1.011(0.243)	0.553	M
Citramalic acid	GC-MS	1.076(0.465)	1.007(0.348)	1.122(0.413)	0.790	T	0.734(0.852)	0.558	M
Citric acid	GC-MS	0.923(0.486)	0.875(0.454)	0.987(0.457)	0.325	M	0.890(0.464)	0.515	M
Creatinine	GC-MS	0.767(1.418)	0.269(0.212)	0.444(0.634)	0.169	M	0.332(0.209)	0.697	M
Cycloleucine	GC-MS	0.564(0.960)	0.371(0.230)	0.593(0.602)	0.105	M	0.321(0.328)	0.721	M
Cytidine	GC-MS	0.864(0.295)	0.905(0.498)	0.969(0.501)	0.280	M	0.816(0.337)	0.660	T
delta15-11-NeuroF	LC-MS/MS (-)	1.030(0.619)	0.519(1.118)	0.519(1.096)	0.769	M	0.519(1.224)	0.801	M
Dihydrotachysterol	LC-MS/MS(+)	0.878(0.520)	0.822(0.411)	0.781(0.425)	0.681	M	0.851(0.477)	0.484	M
Dimethyldithiophosphate	LC-MS/MS(+)	0.919(0.156)	0.899(0.120)	0.898(0.390)	0.570	M	0.819(0.194)	0.171	M
Docosahexaenoic acid	LC-MS/MS(+)	0.638(0.485)	0.506(0.345)	0.972(1.085)	0.246	M	0.357(0.481)	0.171	M

Metabolite	Method	Patient Group		Treatment Group			Healthy Group		
		Mean	Median	Mean (SD) or Median (IQR)	p value	Test*	Mean (SD) or Median (IQR)	p value	Test*
Docosahexanoic acid	LC-MS (-)	1.023(0.681)	0.548(1.163)	0.548(0.000)	0.340	M	0.548(1.158)	0.807	M
Edetic Acid	LC-MS/MS(+)	0.910(0.869)	0.633(0.771)	0.503(0.752)	0.544	M	0.604(0.678)	0.802	M
Ethyl aconitate	LC-MS/MS(+)	0.566(0.167)	0.565(0.210)	0.593(0.319)	0.544	M	0.512(0.351)	0.484	M
Fructose	GC-MS	1.207(0.415)	1.137(0.185)	0.883(0.230)	0.003	M	0.847(0.231)	0.000	M
Fucose	GC-MS	0.484(0.668)	0.224(0.433)	0.325(0.258)	0.905	M	0.098(0.141)	0.024	M
Fumaric acid	GC-MS	0.928(0.159)	0.878(0.185)	1.042(0.197)	0.112	T	0.978(0.259)	0.485	T
Galacturonic acid	GC-MS	0.826(0.650)	0.519(0.448)	0.643(0.362)	0.375	M	0.397(0.187)	0.013	M
Geranyl acetoacetate	LC-MS/MS(+)	0.782(0.321)	0.744(0.361)	0.814(0.253)	0.444	M	0.648(0.263)	0.626	M
Glucoheptonic acid	GC-MS	0.922(0.079)	0.925(0.090)	0.871(0.201)	0.981	M	1.031(0.218)	0.037	T
Gluconic acid	GC-MS	1.516(2.216)	0.538(0.556)	0.631(0.331)	0.981	M	0.400(0.266)	0.055	M
Glucosamine phosphate	GC-MS	0.280(0.191)	0.200(0.161)	0.251(0.158)	0.720	M	1.697(1.022)	0.000	M
Glucosaminic acid	GC-MS	0.774(0.365)	0.636(0.389)	0.897(0.716)	0.185	M	0.550(0.544)	0.537	M
Glucose-6-phosphate	GC-MS	0.782(0.380)	0.787(0.423)	0.472(0.273)	0.024	T	1.542(1.856)	0.028	M
Glutaminyl-Gamma-glutamate	LC-MS/MS(+)	0.379(0.423)	0.231(0.402)	0.257(0.215)	0.765	M	0.196(0.333)	0.667	M
Glutamylthreonine	LC-MS (-)	0.989(0.074)	1.000(0.068)	1.004(0.147)	0.728	T	1.061(0.106)	0.028	M
Glyceraldehyde	GC-MS	1.230(0.286)	1.240(0.549)	1.120(0.338)	0.370	T	0.849(0.279)	0.001	T
Glyceric acid	GC-MS	0.604(0.210)	0.525(0.290)	0.768(0.308)	0.118	T	1.310(0.454)	0.000	M
Glycerol	GC-MS	0.742(0.338)	0.669(0.347)	0.990(0.369)	0.033	M	0.985(0.243)	0.006	M
Glycerol 1-hexadecanoate	LC-MS(+)	1.408(2.537)	0.573(0.411)	0.489(0.347)	0.625	M	0.503(0.529)	0.782	M
Glycerol-phosphate	GC-MS	0.711(0.151)	0.765(0.292)	0.960(0.327)	0.018	T	1.009(0.607)	0.024	M
Glycochenodeoxycholic acid	LC-MS (-)	1.251(1.320)	0.402(1.091)	0.402(1.557)	0.983	M	0.402(0.584)	0.273	M
Glycocholic acid	LC-MS (-)	3.299(3.789)	1.659(4.046)	0.097(0.317)	0.000	M	0.311(0.391)	0.001	M
Glycolic acid	GC-MS	0.723(0.258)	0.670(0.236)	0.685(0.365)	0.720	M	1.255(0.435)	0.000	M
glycoursodeoxycholic acid	LC-MS/MS (-)	2.273(1.835)	2.233(2.820)	0.223(0.312)	0.001	M	0.759(1.036)	0.039	M
Guanidine	LC-MS(+)	0.758(0.652)	0.513(0.496)	1.067(0.611)	0.077	M	1.011(0.412)	0.007	M

Metabolite	Method	Patient Group		Treatment Group			Healthy Group		
		Mean	Median	Mean (SD) or Median (IQR)	p value	Test*	Mean (SD) or Median (IQR)	p value	Test*
Guanidinosuccinic acid	GC-MS	1.525(1.141)	1.258(0.773)	0.740(0.308)	0.038	M	0.577(0.387)	0.002	M
Heptadecanoic acid	GC-MS	0.906(0.342)	0.880(0.476)	1.138(0.431)	0.132	T	0.919(0.373)	0.267	M
Hippuric acid	GC-MS	0.373(0.644)	0.098(0.396)	0.262(0.318)	0.220	M	0.069(0.073)	0.344	M
Histidinyl-Isoleucine	LC-MS/MS(+)	0.708(1.373)	0.000(0.355)	0.000(0.000)	0.158	M	0.000(0.199)	0.436	M
Homodeoxycholic acid	LC-MS/MS (-)	1.038(0.193)	1.010(0.082)	1.063(0.287)	0.544	M	1.023(0.094)	0.589	M
Homoserine	GC-MS	0.465(0.192)	0.407(0.217)	0.541(0.241)	0.085	M	1.222(1.304)	0.009	M
Hydroquinone	GC-MS	1.012(0.095)	1.025(0.147)	0.995(0.054)	0.578	T	0.978(0.067)	0.272	T
Hyoscyamine	LC-MS(+)	0.715(0.970)	0.248(0.292)	0.212(0.620)	0.681	M	0.175(0.166)	0.016	M
Hypotaurine	GC-MS	0.383(0.145)	0.360(0.118)	2.062(1.471)	0.000	M	0.355(0.158)	0.974	M
Iminodiacetic acid	GC-MS	0.674(0.096)	0.658(0.114)	0.722(0.907)	0.094	M	0.741(0.297)	0.312	M
Indole-3-carbinol	LC-MS(+)	1.032(0.996)	0.885(0.820)	1.320(1.957)	0.316	M	0.473(0.752)	0.153	M
Indole-3-methyl acetate	LC-MS (-)	1.171(1.048)	0.716(0.781)	0.974(1.540)	0.541	M	0.477(0.000)	0.030	M
Indole-3-propionic acid	LC-MS/MS(+)	1.073(1.073)	0.876(1.018)	1.019(1.924)	0.392	M	0.435(0.810)	0.102	M
Isocitric acid	GC-MS	0.728(0.144)	0.758(0.086)	0.796(1.315)	0.402	M	0.923(0.258)	0.006	M
Isomaltose	GC-MS	0.924(0.090)	0.941(0.132)	1.092(0.161)	0.003	T	1.033(0.162)	0.014	T
Isopalmitic acid	LC-MS (-)	0.980(0.751)	1.044(0.826)	1.027(0.872)	0.792	M	0.915(0.687)	0.773	M
Itaconic acid	GC-MS	1.069(1.275)	0.561(0.707)	1.001(0.833)	0.280	M	0.557(0.420)	0.434	M
L-2-Amino-4-methylenepentanedioic acid	LC-MS/MS(+)	0.901(0.354)	0.927(0.451)	0.790(0.385)	0.413	T	0.735(0.472)	0.501	M
Lactamide	GC-MS	0.986(1.010)	0.538(1.058)	0.517(0.305)	0.905	M	0.625(1.574)	0.795	M
Lactic acid	GC-MS	0.710(0.240)	0.659(0.106)	0.820(0.320)	0.323	T	1.186(0.546)	0.000	M
Lactose	GC-MS	1.127(0.213)	1.055(0.100)	0.992(0.143)	0.128	M	0.994(0.261)	0.016	M
L-Alanine	GC-MS	0.745(0.630)	0.376(0.847)	0.824(1.181)	0.375	M	0.749(0.611)	0.673	M
Lauric acid	GC-MS	1.013(0.896)	0.807(1.061)	0.486(0.258)	0.756	M	1.058(0.745)	0.169	M
L-beta-aspartyl-L-glutamic acid	LC-MS/MS(+)	0.303(0.701)	0.000(0.117)	0.038(0.206)	0.268	M	0.141(0.489)	0.077	M
L-beta-aspartyl-L-threonine	LC-MS(+)	0.880(0.405)	0.819(0.540)	0.760(0.296)	0.570	M	0.683(0.310)	0.308	M

Metabolite	Method	Patient Group		Treatment Group			Healthy Group		
		Mean	Median	Mean (SD) or Median (IQR)	p value	Test*	Mean (SD) or Median (IQR)	p value	Test*
L-Glutamic acid	GC-MS	1.157(0.457)	1.172(0.893)	0.757(0.237)	0.302	M	0.958(0.483)	0.580	M
L-Glutamine	LC-MS (-)	1.111(0.521)	1.309(1.047)	1.187(0.993)	0.606	M	1.221(0.941)	0.497	M
L-Glycine	GC-MS	0.880(0.299)	0.845(0.392)	1.077(0.471)	0.038	M	0.743(0.282)	0.192	T
Linoleic acid	GC-MS	0.865(0.619)	0.645(1.092)	1.157(0.491)	0.189	T	1.062(0.518)	0.341	T
L-Isoleucine	GC-MS	0.929(0.342)	0.942(0.376)	1.031(0.441)	0.507	T	0.911(0.418)	0.948	M
L-Leucine	GC-MS	0.902(0.367)	0.909(0.472)	1.005(0.417)	0.500	T	1.003(0.413)	0.494	M
L-Lysine	GC-MS	0.778(0.348)	0.754(0.254)	0.923(0.610)	0.169	M	0.948(0.525)	0.253	T
L-Methionine	GC-MS	0.902(0.397)	0.763(0.576)	0.833(0.576)	0.430	M	0.770(0.518)	0.649	M
LPC 16:0	LC-MS/MS (-)	1.016(0.436)	1.108(0.643)	1.042(0.271)	0.850	T	0.870(0.383)	0.535	M
LPC 18:1	LC-MS/MS (-)	1.080(0.735)	0.950(1.170)	1.184(0.511)	0.149	M	0.710(0.467)	0.484	M
LPC 18:2	LC-MS/MS (-)	0.846(0.465)	0.837(0.501)	1.218(0.535)	0.048	T	1.013(0.416)	0.268	T
LPE 16:0	LC-MS (-)	1.506(1.319)	1.061(1.028)	1.129(0.638)	0.921	M	0.779(0.582)	0.065	M
LPE 18:2	LC-MS (-)	1.074(0.829)	0.992(0.859)	1.394(1.050)	0.109	M	0.758(0.526)	0.864	M
L-Phenylalanine	GC-MS	1.053(0.348)	0.894(0.488)	0.911(0.486)	0.867	M	0.875(0.459)	0.226	M
L-Pipecolic acid	LC-MS(+)	0.572(0.165)	0.554(0.210)	0.575(0.234)	0.653	M	0.557(0.166)	0.722	M
L-Proline	GC-MS	1.019(0.433)	0.934(0.491)	1.003(0.463)	0.928	T	0.908(0.425)	0.474	M
L-Threonine	GC-MS	0.845(0.310)	0.812(0.551)	0.961(0.373)	0.389	T	1.000(0.424)	0.220	T
L-trans-alpha-Amino-2-carboxycyclopropaneacetic acid	LC-MS/MS(+)	0.975(0.313)	0.907(0.360)	0.907(0.344)	0.568	T	0.898(0.431)	0.702	M
L-Tryptophan	GC-MS	0.207(0.159)	0.147(0.235)	0.148(0.331)	0.905	M	0.252(0.515)	0.415	M
L-Tyrosine	GC-MS	0.775(0.409)	0.760(0.567)	0.929(0.547)	0.412	T	0.890(0.811)	0.123	M
L-Valine	GC-MS	0.895(0.364)	0.957(0.454)	0.998(0.312)	0.439	T	0.958(0.379)	0.415	M
LysoPC(16:0)	LC-MS/MS(+)	0.432(1.507)	0.026(0.034)	0.022(0.062)	0.922	M	0.035(0.081)	0.321	M
LysoPC(17:0)	LC-MS/MS(+)	0.757(0.587)	0.633(0.430)	1.041(0.688)	0.029	M	0.608(0.485)	0.947	M
LysoPE(0:0/20:2(11Z,14Z))	LC-MS/MS (-)	0.896(0.421)	0.895(0.391)	1.220(0.478)	0.055	T	1.056(0.377)	0.242	T
LysoPE(18:0/0:0)	LC-MS/MS (-)	1.080(0.475)	1.125(0.694)	1.097(0.301)	0.910	T	0.912(0.391)	0.626	M

Metabolite	Method	Patient Group		Treatment Group			Healthy Group		
		Mean	Median	Mean (SD) or Median (IQR)	p value	Test*	Mean (SD) or Median (IQR)	p value	Test*
Lyxose	GC-MS	1.333(0.567)	1.222(0.415)	0.905(0.458)	0.105	M	0.600(0.223)	0.001	T
Lyxosylamine	GC-MS	0.946(0.646)	0.924(0.627)	0.540(0.256)	0.583	M	0.584(0.435)	0.149	M
Malic acid	GC-MS	0.804(0.289)	0.781(0.108)	1.070(0.566)	0.259	M	0.949(0.496)	0.040	M
Malonic acid	LC-MS/MS (-)	1.079(0.121)	1.073(0.197)	1.054(0.106)	0.545	T	1.084(0.108)	0.898	T
Maltose	GC-MS	0.316(0.113)	0.331(0.074)	0.467(0.168)	0.007	M	0.413(0.126)	0.036	M
Maltotriose	GC-MS	0.002(0.002)	0.001(0.002)	0.010(0.018)	0.038	M	0.008(0.017)	0.267	M
Mannitol	GC-MS	1.595(1.188)	1.438(1.547)	0.382(0.591)	0.014	M	0.761(0.574)	0.031	T
Mannose	GC-MS	0.985(0.040)	1.001(0.046)	1.019(0.054)	0.074	T	0.978(0.073)	0.696	T
Melibiose	GC-MS	0.305(0.073)	0.290(0.069)	0.451(0.142)	0.003	T	0.407(0.243)	0.020	M
Methyl Heptadecanoic acid	LC-MS (-)	0.657(0.710)	0.230(0.720)	0.854(1.230)	0.214	M	1.055(1.556)	0.066	M
Methyl palmitate	GC-MS	0.965(0.281)	0.910(0.124)	0.979(0.152)	0.302	M	1.006(0.201)	0.179	M
Methyl stearate	GC-MS	0.762(0.403)	0.704(0.272)	0.915(0.311)	0.284	T	1.021(1.341)	0.100	M
Methyl-beta-D-galactopyranoside	GC-MS	1.274(1.062)	0.891(0.810)	0.775(0.383)	0.650	M	0.635(0.790)	0.397	M
Methylmalonic acid	GC-MS	2.767(7.753)	0.697(0.753)	0.522(0.592)	0.583	M	0.314(0.373)	0.075	M
MG(0:0/16:0/0:0)	LC-MS/MS(+)	1.029(0.899)	0.954(0.750)	0.804(1.204)	0.710	M	0.777(1.052)	0.683	M
MG(0:0/20:3(5Z,8Z,11Z)/0:0)	LC-MS/MS(+)	1.046(0.481)	0.876(0.330)	0.825(0.189)	0.421	M	0.831(0.461)	0.702	M
MG(0:0/22:5(7Z,10Z,13Z,16Z,19Z)/0:0)	LC-MS/MS (-)	0.786(0.410)	0.537(0.594)	0.537(1.124)	0.142	M	0.537(0.914)	0.265	M
MG(18:0/0:0/0:0)	LC-MS/MS(+)	0.878(1.868)	0.086(0.185)	0.053(0.068)	0.128	M	0.085(0.128)	0.989	M
MG(18:3(6Z,9Z,12Z)/0:0/0:0)	LC-MS/MS(+)	1.209(1.777)	0.626(0.524)	0.421(0.362)	0.597	M	0.575(0.677)	0.683	M
MG(20:3(11Z,14Z,17Z)/0:0/0:0)	LC-MS/MS(+)	0.967(0.810)	0.678(0.609)	0.958(0.317)	0.399	M	0.931(0.537)	0.189	M
MG(20:3(5Z,8Z,11Z)/0:0/0:0)	LC-MS/MS(+)	0.949(0.580)	0.824(0.624)	0.709(0.499)	0.625	M	0.886(0.566)	0.722	M
Myo-inositol	GC-MS	0.847(0.372)	1.039(0.690)	1.009(0.107)	0.867	M	1.028(0.187)	0.361	M
Myristic acid	GC-MS	0.979(0.597)	0.771(0.926)	0.676(0.463)	0.793	M	1.001(0.336)	0.239	M
N-(2-hydroxyethyl)iminodiacetic acid	GC-MS	0.582(0.090)	0.581(0.086)	0.711(0.423)	0.043	M	0.836(0.614)	0.005	M
N,n-dimethylglycine	GC-MS	1.050(0.091)	1.041(0.070)	0.997(0.049)	0.169	M	0.964(0.046)	0.002	M

Metabolite	Method	Patient Group		Treatment Group			Healthy Group		
		Mean	Median	Mean (SD) or Median (IQR)	p value	Test*	Mean (SD) or Median (IQR)	p value	Test*
N-acetyl-5-hydroxytryptamine	GC-MS	0.837(0.087)	0.828(0.126)	1.061(0.343)	0.032	T	1.078(0.301)	0.001	M
N-acetyl-d-mannosamine	GC-MS	1.250(1.165)	1.086(0.871)	1.470(1.818)	0.458	M	0.415(0.736)	0.037	M
N-acetyl-l-aspartic acid	GC-MS	0.673(0.156)	0.643(0.233)	0.728(0.348)	0.116	M	0.762(0.291)	0.454	M
N-acetyl-l-cysteine	GC-MS	0.776(0.384)	0.730(0.596)	1.249(0.745)	0.050	T	0.574(0.702)	0.948	M
N-acetyl-l-glutamic acid	GC-MS	1.072(0.285)	1.125(0.462)	0.943(0.254)	0.227	T	1.125(0.265)	0.587	T
N-ethylglycine	GC-MS	0.764(0.388)	0.636(0.227)	0.898(0.347)	0.358	T	1.021(0.663)	0.026	M
N-gamma-Acetyl-N-2-formyl-5-methoxykynurenamine	LC-MS (-)	1.649(3.370)	0.237(1.180)	0.512(0.841)	0.513	M	0.134(0.000)	0.000	M
N-methylalanine	GC-MS	0.450(0.259)	0.410(0.376)	0.990(0.616)	0.008	T	0.766(0.843)	0.040	M
N-methyl-glutamic acid	GC-MS	0.706(0.080)	0.724(0.066)	0.794(1.578)	0.202	M	0.662(0.271)	0.697	M
N-Methyllysine	LC-MS (-)	1.015(0.085)	1.007(0.099)	1.052(0.140)	0.376	T	1.099(0.096)	0.007	T
Norhyocholic acid	LC-MS/MS(+)	0.919(0.398)	0.875(0.623)	0.918(0.326)	0.993	T	0.893(0.379)	0.842	T
Octadecanamide	LC-MS/MS(+)	0.891(0.662)	0.745(0.630)	0.608(0.727)	0.953	M	0.542(0.920)	0.607	M
Octadecanedioic acid	LC-MS(+)	1.352(2.125)	0.671(1.031)	1.053(1.151)	0.421	M	0.315(0.616)	0.249	M
Oleamide	LC-MS/MS(+)	0.563(1.097)	0.000(0.461)	0.000(0.065)	0.981	M	0.173(0.497)	0.457	M
Oleic acid	GC-MS	0.879(0.636)	0.759(1.113)	1.177(0.606)	0.226	T	1.017(0.532)	0.515	T
O-phosphocolamine	GC-MS	0.545(0.352)	0.360(0.468)	1.107(0.297)	0.000	M	1.112(0.757)	0.005	M
Orotic acid	GC-MS	0.140(0.104)	0.103(0.081)	0.075(0.023)	0.003	M	0.068(0.033)	0.000	M
Oxalacetic acid	GC-MS	0.883(0.114)	0.870(0.136)	1.021(0.138)	0.033	M	0.899(0.221)	0.434	M
Oxalic acid	GC-MS	0.710(0.346)	0.677(0.433)	1.146(0.482)	0.012	T	0.830(0.529)	0.328	M
Palmitic acid	GC-MS	0.846(0.229)	0.806(0.362)	1.063(0.300)	0.044	T	1.061(0.256)	0.015	T
Palmitic amide	LC-MS/MS(+)	0.653(0.451)	0.568(0.495)	0.744(1.287)	0.444	M	0.821(0.840)	0.296	M
Palmitoleic acid	GC-MS	0.937(0.786)	0.808(1.343)	0.676(1.098)	0.830	M	0.715(0.615)	0.649	M
p-Aminobenzoic acid	LC-MS(+)	0.968(0.872)	0.756(0.323)	0.788(0.252)	0.399	M	0.879(0.335)	0.155	M
PC(18:2(9Z,12Z)/20:5(5Z,8Z,11Z,14Z,17Z))	LC-MS/MS (-)	0.879(0.864)	0.517(0.000)	0.517(0.517)	0.553	M	0.517(0.620)	0.692	M
PC(O-17:0/20:4(5Z,8Z,11Z,14Z))	LC-MS/MS(+)	1.142(1.394)	0.462(1.384)	0.850(0.866)	0.681	M	0.471(1.648)	0.968	M

Metabolite	Method	Patient Group		Treatment Group			Healthy Group		
		Mean	Median	Mean (SD) or Median (IQR)	p value	Test*	Mean (SD) or Median (IQR)	p value	Test*
P-cresol	GC-MS	1.008(0.841)	0.699(0.971)	1.046(0.453)	0.220	M	0.365(0.181)	0.007	M
PE(O-18:0/22:4(7Z,10Z,13Z,16Z))	LC-MS/MS(+)	1.117(1.223)	0.479(1.459)	0.890(0.891)	1.000	M	0.465(1.525)	0.864	M
PE(O-18:1(9Z)/0:0)	LC-MS/MS(+)	2.874(2.645)	2.072(3.493)	0.133(0.509)	0.000	M	0.311(0.362)	0.000	M
PE(P-18:0/20:1(11Z))	LC-MS/MS(+)	1.233(1.270)	0.836(1.748)	1.005(1.093)	0.769	M	0.515(0.901)	0.405	M
PFCA-H	LC-MS (-)	1.129(0.423)	1.095(0.763)	0.724(0.000)	0.053	M	0.724(0.784)	0.804	M
PG(16:0/18:1(11Z))	LC-MS/MS (-)	0.733(0.537)	0.589(0.000)	0.589(0.000)	0.292	M	0.589(0.000)	0.405	M
Phenylacetaldehyde	GC-MS	1.181(0.261)	1.128(0.276)	0.934(0.552)	0.150	T	0.996(0.209)	0.159	M
Phenyl-beta-glucopyranoside	GC-MS	0.860(1.278)	0.250(0.348)	1.021(1.090)	0.029	M	0.180(0.177)	0.415	M
Phenylethylamine	GC-MS	0.016(0.006)	0.015(0.007)	1.208(3.193)	0.000	M	0.018(0.009)	0.239	M
Phosphatidylcholine lyso 16	LC-MS (-)	1.089(0.903)	0.792(1.271)	1.044(0.956)	0.855	M	0.339(0.575)	0.232	M
Phosphatidylethanolamine lyso 18	LC-MS (-)	1.494(1.450)	1.295(1.198)	1.335(0.490)	0.605	M	0.429(0.107)	0.003	M
Phosphatidylethanolamine lyso 20	LC-MS (-)	1.110(0.647)	1.140(0.592)	1.187(0.311)	0.689	T	0.876(0.322)	0.292	M
Phosphoric acid	GC-MS	0.745(0.199)	0.736(0.246)	0.972(0.289)	0.025	T	1.208(0.341)	0.000	T
Phosphorylcholine	LC-MS(+)	0.684(0.276)	0.684(0.362)	1.041(0.622)	0.015	M	0.789(0.495)	0.133	M
Picolonic acid	GC-MS	0.973(0.109)	0.997(0.193)	1.087(0.239)	0.123	T	0.942(0.165)	0.499	T
Pimelic acid	GC-MS	0.703(0.449)	0.633(0.247)	0.824(0.813)	0.105	M	0.392(0.292)	0.047	M
Pipecolic acid	GC-MS	1.136(1.479)	0.525(0.421)	0.947(0.377)	0.094	M	0.789(0.324)	0.281	M
Porphobilinogen	LC-MS (-)	0.536(0.447)	0.408(0.334)	0.567(0.340)	0.462	M	0.573(0.391)	0.400	M
Proline betaine	LC-MS(+)	0.458(1.307)	0.063(0.158)	0.218(0.412)	0.161	M	0.075(0.469)	0.260	M
PS(18:0/20:5(5Z,8Z,11Z,14Z,17Z))	LC-MS/MS(+)	0.570(0.526)	0.342(0.788)	0.274(0.491)	0.619	M	0.625(0.683)	0.245	M
Purine riboside	GC-MS	1.069(1.195)	0.678(0.336)	0.593(0.234)	0.830	M	0.474(0.255)	0.009	M
Pyroglutamic acid	GC-MS	0.882(0.157)	0.865(0.291)	1.043(0.292)	0.087	T	0.927(0.386)	0.626	T
Pyruvic acid	GC-MS	0.880(0.181)	0.854(0.229)	1.122(0.295)	0.017	T	0.932(0.244)	0.468	T
Ribitol	GC-MS	0.713(0.208)	0.678(0.198)	0.660(0.331)	0.867	M	0.647(0.218)	0.380	T
Ribonic acid-gamma-lactone	GC-MS	0.491(0.455)	0.366(0.457)	0.169(0.208)	0.583	M	0.147(0.058)	0.010	M

Metabolite	Method	Patient Group		Treatment Group			Healthy Group		
		Mean	Median	Mean (SD) or Median (IQR)	p value	Test*	Mean (SD) or Median (IQR)	p value	Test*
Ribose	GC-MS	0.873(0.085)	0.860(0.143)	0.948(0.122)	0.043	M	0.981(0.133)	0.000	M
Saccharic acid	GC-MS	0.941(0.976)	0.448(1.320)	0.662(0.289)	0.220	M	0.241(0.084)	0.001	M
Sarcosine	GC-MS	1.309(1.652)	0.686(1.509)	0.336(0.883)	0.488	M	0.390(1.579)	0.745	M
Sedoheptulose	GC-MS	0.805(1.066)	0.256(0.766)	0.467(0.456)	0.402	M	0.549(1.124)	0.845	M
Squalene	GC-MS	0.732(1.419)	0.354(0.187)	0.642(0.596)	0.141	M	0.580(0.653)	0.034	M
Stearic acid	GC-MS	0.844(0.117)	0.826(0.178)	0.985(0.191)	0.011	M	0.966(0.251)	0.000	M
Succinic acid	GC-MS	0.781(0.139)	0.811(0.156)	1.013(0.194)	0.002	T	1.060(0.321)	0.001	T
Sucrose	GC-MS	0.686(0.870)	0.200(0.532)	0.267(0.419)	0.981	M	0.252(0.193)	0.603	M
Tartaric acid	GC-MS	0.205(0.390)	0.108(0.088)	0.440(1.084)	0.000	M	0.075(0.073)	0.494	M
Tartronic acid	GC-MS	1.067(0.982)	0.531(0.536)	0.689(0.460)	0.430	M	0.616(0.329)	0.580	M
Taurocholic acid	LC-MS/MS (-)	1.439(1.221)	1.098(1.373)	0.686(1.198)	0.379	M	0.343(0.715)	0.020	M
Testosterone	GC-MS	0.565(0.850)	0.154(0.086)	0.121(1.750)	0.830	M	1.658(2.055)	0.296	M
Tetracosanoic acid	LC-MS(+)	1.035(1.569)	0.217(0.708)	0.123(0.606)	0.625	M	0.508(1.721)	0.484	M
Threitol	GC-MS	1.154(0.982)	0.715(0.970)	0.696(0.395)	0.720	M	0.481(0.150)	0.001	M
Threonic acid	LC-MS (-)	1.259(0.680)	0.964(0.747)	0.752(0.308)	0.109	M	0.834(0.350)	0.133	M
Threose	GC-MS	0.728(0.232)	0.752(0.400)	1.209(0.544)	0.007	T	0.668(0.347)	0.974	M
Tiglylcarnitine	LC-MS/MS (-)	0.885(0.181)	0.921(0.166)	1.080(0.273)	0.024	T	1.030(0.383)	0.126	M
Trans-aconitic acid	GC-MS	0.834(0.842)	0.570(0.506)	0.741(0.498)	0.325	M	0.466(0.295)	0.397	M
Trehalose-6-phosphate	GC-MS	0.236(0.072)	0.242(0.083)	0.216(0.108)	0.905	M	0.233(0.083)	0.745	M
Tryptophanol	LC-MS(+)	0.970(0.378)	1.020(0.559)	0.952(0.322)	0.887	T	1.013(0.671)	0.376	M
Uracil	GC-MS	0.348(0.221)	0.410(0.342)	0.563(0.362)	0.073	T	0.462(0.651)	0.454	M
Urea	GC-MS	0.645(0.722)	0.050(1.175)	1.064(1.540)	0.350	M	1.171(1.392)	0.494	M
Uric acid	LC-MS (-)	0.859(0.779)	0.500(0.928)	0.642(0.523)	0.860	M	1.099(0.353)	0.021	M
Xylitol	GC-MS	1.132(1.018)	0.674(0.834)	0.697(0.449)	0.793	M	0.531(0.223)	0.009	M

T: The groups which follow normal distribution were compared by two-tailed Student's t-test. M: Nonnormally distributed groups were carried out by Mann Whitney U test. SD: Standard deviation. IQR: Interquartile range

Table S1.2. Statistical analysis results of metabolites involved in sulfur metabolism identified by LC-MS/MS from plasma samples.

Metabolite	Patient Group	Treatment group		Healthy Group	
	Mean* ± SE	Mean* ± SE	p	Mean* ± SE	p
**Serine**	0.482 ± 0.060	0.553 ± 0.062	0.436	3.291 ± 0.245	0.000
**Cysteine**	1.035 ± 0.018	1.050 ± 0.022	0.631	1.022 ± 0.008	0.437
**Taurine**	0.645 ± 0.403	3.110 ± 0.626	0.005	13.704 ± 1.476	0.000
**Glutathione**	0.073 ± 0.019	0.098 ± 0.016	0.335	0.156 ± 0.010	0.000
**Cystine**	0.097 ± 0.045	0.150 ± 0.061	0.513	0.053 ± 0.019	0.317

* All results are in ppm units.

The data obtained from GC-MS, LC-qTOF-MS and LC-MS/MS were processed with multivariant analyses and PCA graphs were presented in **Figure S1.1.** PLS-DA graphs were obtained to evaluate differences between groups, VIP (Variable Important in Project) graphs showing the most important metabolites leading differences in data analysis, R^2 (the fraction of variance explained by a component) and Q^2 (the fraction of the total variation predicted by a component) values to determine the confidence of PLS-DA and coefficient graphs to determine increasing and decreasing metabolites between groups. We saw that metabolomics profiles were completely different between study groups that differentiate each other **(Figure S1.2-4).**

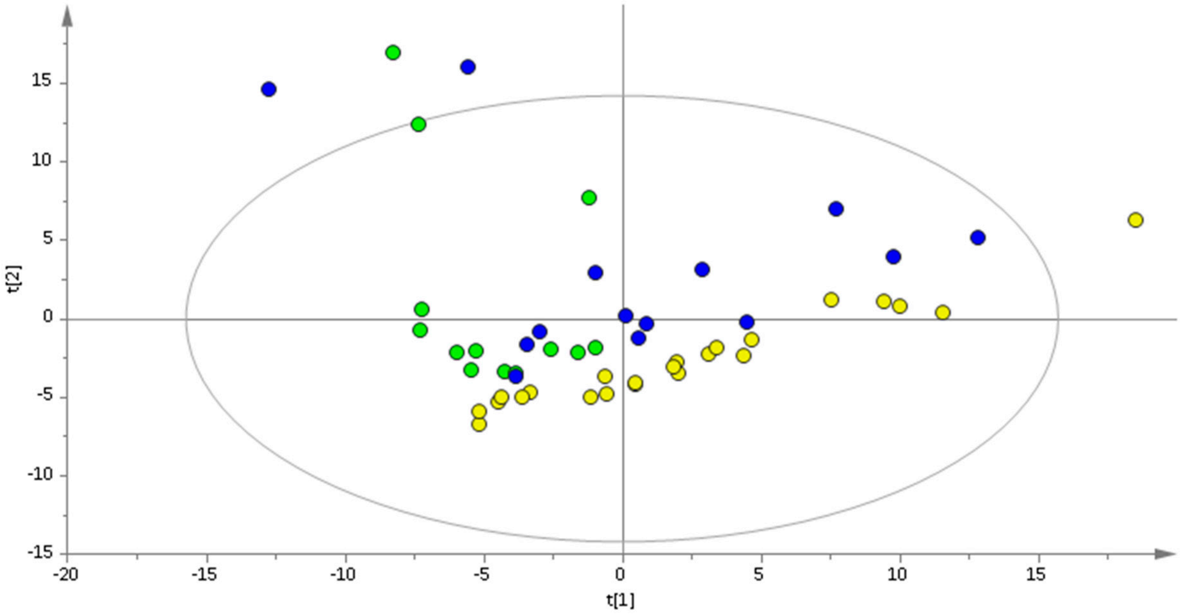

Figure S1.1. PCA graph of metabolomics analyses of plasma samples through GC-MS, LC-qTOF-MS and LC-MS/MS (yellow dots: healthy control; blue dots: treatment; green dots: patients)

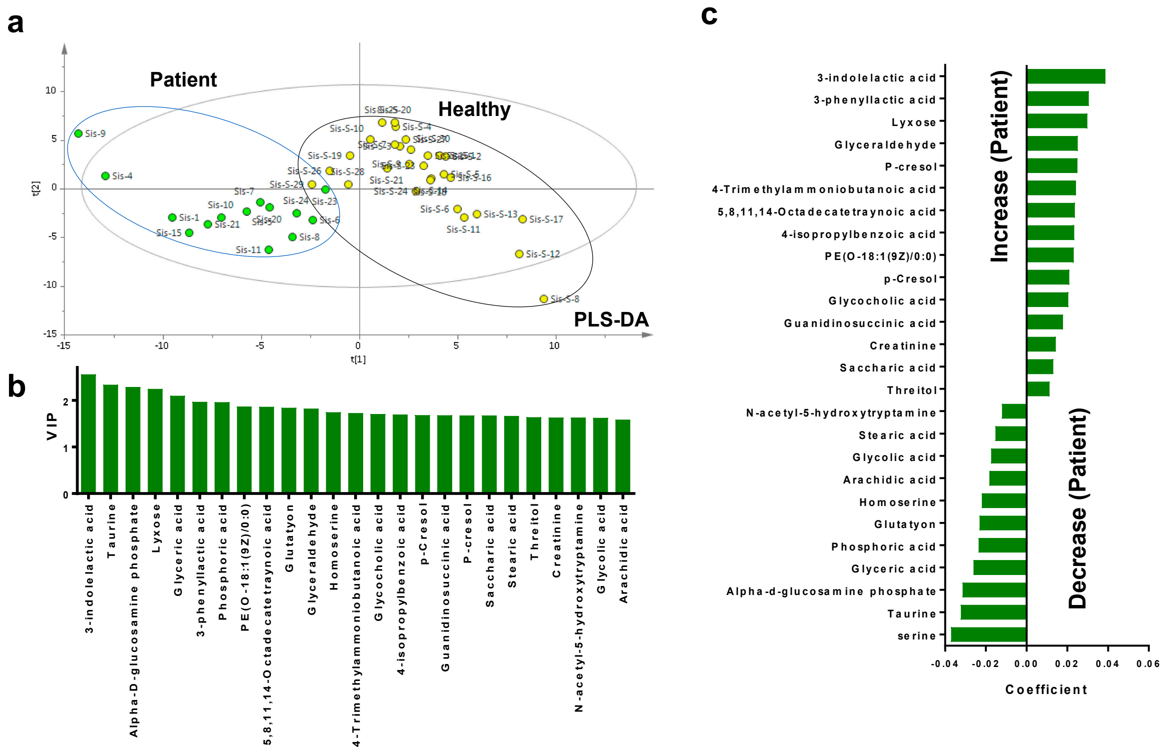

Figure S1.2. Multivariate analyses of data obtained from plasma metabolomics analysis by GC-MS, LC-qTOF-MS and LC-MS/MS (patient group vs healthy group). **a)** PLS-DA score graph (R^2: 0.839, Q^2: 0.553) **b)** VIP (Variable Important in Project) graph showing most important metabolites leading differences in PLS-DA analysis **c)** Coefficient graph showing increasing and decreasing metabolites between groups

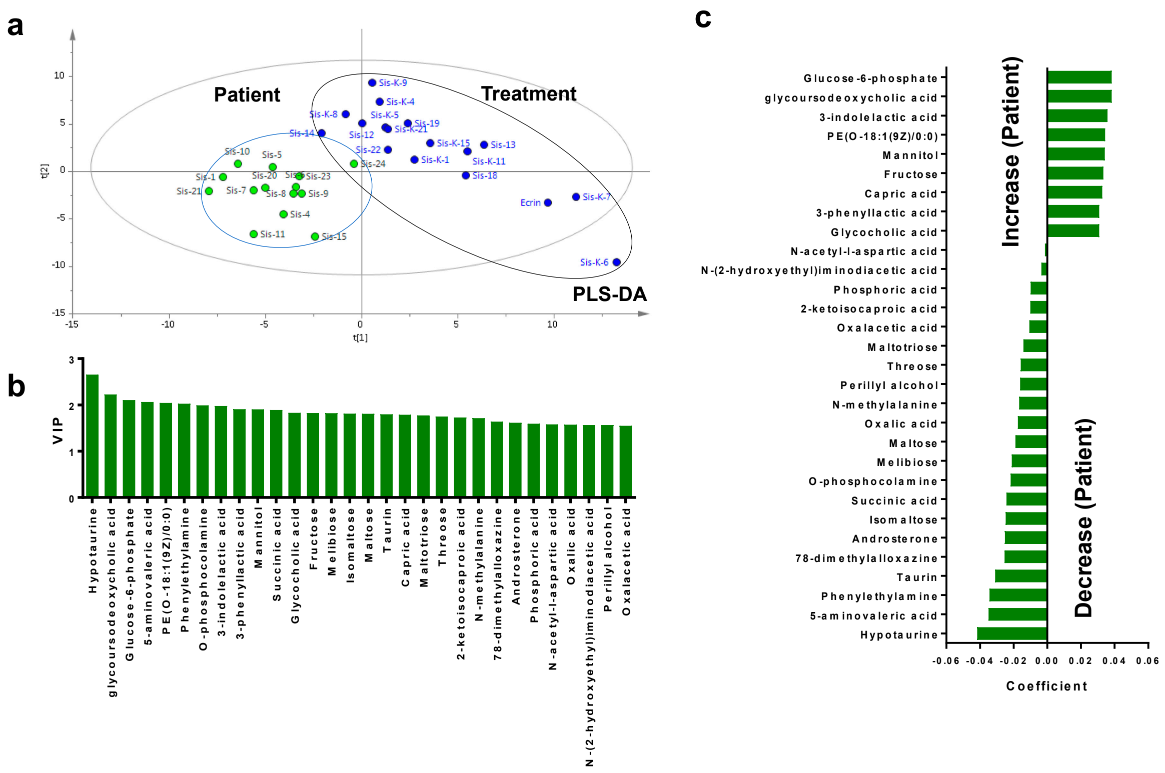

Figure S1.3. Multivariate analyses of data obtained from plasma metabolomics analysis by GC-MS, LC-qTOF-MS and LC-MS/MS (patient group vs treatment group). **a)** PLS-DA score graph (R^2: 0.869, Q^2: 0.399) **b)** VIP graph showing most important metabolites leading differences in data analysis **c)** Coefficient graph showing increasing and decreasing metabolites between groups

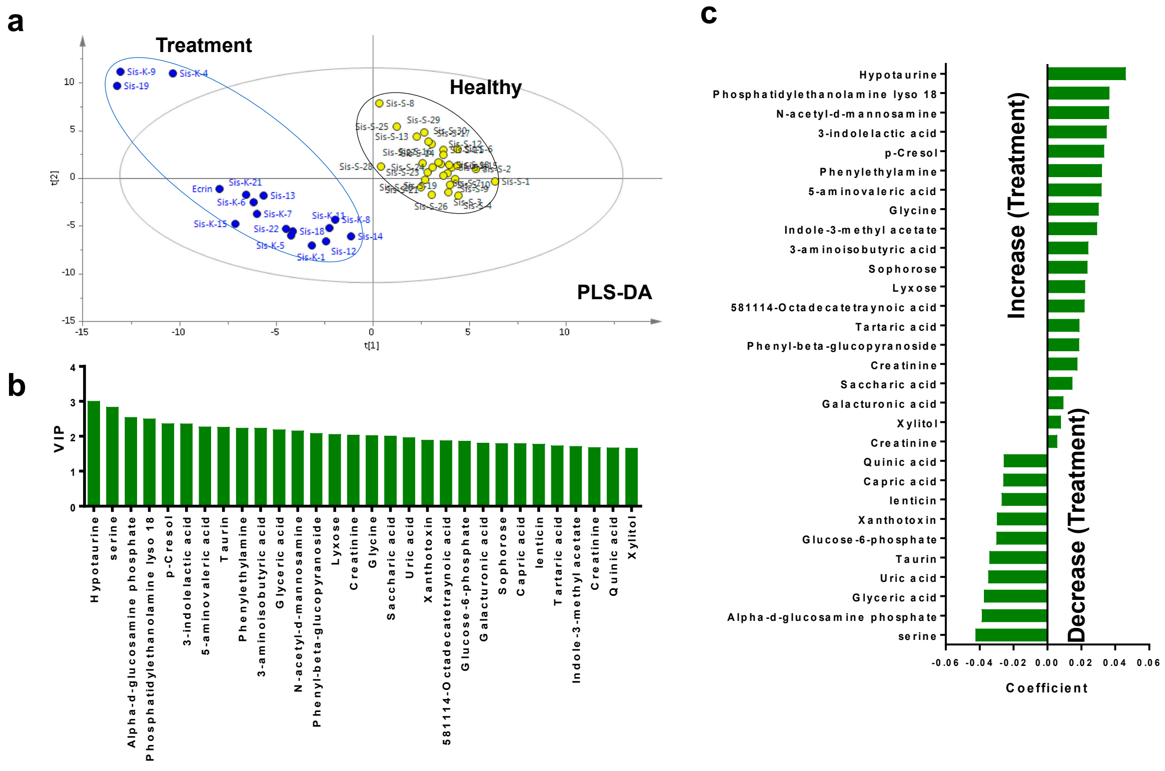

Figure S1.4. Multivariate analyses of data obtained from plasma metabolomics analysis by GC-MS, LC-qTOF-MS and LC-MS/MS (treatment group vs healthy group) **a)** PLS-DA score graph (R^2: 0.942, Q^2: 0.760) **b)** VIP (Variable Important in Project) graph showing most important metabolites leading differences in data analysis **c)** Coefficient graph showing increasing and decreasing metabolites between groups

When results of false classification analyses according to PLS-DA method were evaluated, it was observed that the groups were differentiated from each other with a high sensitivity (>92%). It was even 100% between treatment versus healthy groups. Fischer's false classification probability of PLS-DA method was calculated as $3.8x10^{-10}$, $6.8x10^{-8}$, $5.7x10^{-13}$ for patient versus healthy groups, patients versus treatment group, and treatment versus healthy group, respectively **(Table S1.3)**.

Table S1.3. False classification analyses for plasma samples according to the PLS-DA method

	Members	Correct	Patient	Healthy
**Patient**	14	92.86%	13	1
**Healthy**	29	100%	0	29
Fishers prob.	$3.8x10^{-10}$			

	Members	Correct	Patient	Treatment
**Patient**	14	92.86%	13	1
**Treatment**	17	100%	0	17
Fishers prob.	$6.8x10^{-8}$			

	Members	Correct	Treatment	Healthy
**Tedavi**	17	100%	17	0
**Healthy**	29	100%	0	29
Fishers prob.	$5.7x10^{-13}$			

S1.2. Urine samples

The study included 61 urine samples from 14 patients with newly diagnosed cystinosis, 17 patients with cystinosis patients under treatment, and 30 age- and sex-matched healthy controls. Urine samples could not be collected from the two patients undergoing treatment because the required criteria were not satisfied. Urine samples' metabolomic profiles for the healthy, patients, and treatment groups were examined using GC-MS, LC-qTOF-MS, and LC-MS/MS techniques, similarly to the analysis of plasma samples. 382 features were determined as a result of the GC-MS analysis, and 232 of these were identified using the Fiehn and Golm libraries. As a result of the LC-qTOF-MS analysis, 9632 features in the positive ionization mode and 4872 features in the negative ionization mode were determined, and 110 of them were identified in the positive ionization mode and 34 in the negative ionization mode. The list of metabolites and statistical analysis results were given in **Table S1.4**. While 48 metabolites identified by GC-MS were significantly different between patient and treatment groups, 115 were significantly different between patient and healthy groups. Ten of the metabolites identified by LC-qTOF-MS differed significantly between cystinosis and cystinosis therapy groups, whereas 28 differed significantly between patient and healthy groups. Furthermore, LC-MS/MS analysis of sulfur metabolism metabolites showed significant differences in cystine between patient and treatment groups, as well as cystine, serine, methionine, and glutathione between patient and healthy groups **(Table S1.5).**

Table S1.4. Statistical analysis results of metabolites identified by GC-MS and LC-qTOF-MS based metabolomics analysis from urine samples.

Metabolite	Method	Patient group		Treatment group			Healthy Group		
		Mean	Median	Mean(SD) or Median(IQR)	P value	Test*	Mean(SD) or Median(IQR)	Pvalue	Test*
((1S,9ar)-octahydro-1H-quinolizin-1-yl)methanamine	LC-MS (+)	0.927(0.881)	0.640(0.620)	0.688(0.521)	0.922	M	0.539(0.511)	0.399	M
(+)-Mahanimbine	LC-MS (+)	0.265(0.717)	0.027(0.120)	0.027(0.067)	1.000	M	1.425(2.123)	0.000	M
(2E,4Z)-decadienoylcarnitine	LC-MS/MS (+)	0.654(1.823)	0.038(0.159)	0.030(0.102)	0.762	M	0.385(1.387)	0.002	M
(5Z)-13-carboxytridec-5-enoylcarnitine	LC-MS/MS (+)	0.666(2.299)	0.042(0.109)	0.073(0.083)	0.130	M	0.899(1.137)	0.000	M
(9E)-9-nitrooctadecenoic Acid	LC-MS/MS (+)	0.618(2.158)	0.024(0.052)	0.036(0.078)	1.000	M	0.790(0.803)	0.000	M
(9Z)-3-hydroxydodecenoylcarnitine	LC-MS/MS (+)	0.266(0.815)	0.044(0.053)	0.030(0.152)	0.735	M	1.082(1.350)	0.000	M
(9Z,12Z)-N-Benzyloctadeca-9,12-dienamide	LC-MS (+)	0.912(0.333)	0.869(0.308)	1.136(0.226)	0.044	T	1.060(0.290)	0.134	M
(E)-2-decylpent-2-enedioic acid	LC-MS (+)	1.125(0.476)	0.875(0.663)	0.865(0.472)	0.799	M	0.947(0.574)	0.488	M
(R)-Sulcatol	LC-MS (+)	1.001(0.168)	0.970(0.285)	1.008(0.100)	0.885	T	1.044(0.215)	0.623	M
(Z)-6-methoxybenzofuran-3(2H)-one oxime	LC-MS (+)	0.374(0.633)	0.129(0.170)	0.232(0.291)	0.297	M	0.804(2.212)	0.015	M
[5-hydroxy-3-(hydroxymethyl)-2-oxo-6-propan-2-ylcyclohex-3-en-1-yl] 3-methylpentanoate	LC-MS (+)	0.868(0.073)	0.888(0.079)	0.892(0.046)	0.739	M	0.870(0.096)	0.588	M
1-(2-hydroxy-3-methyl)-butyl-hydrocotarnine	LC-MS (+)	0.480(1.449)	0.043(0.097)	0.068(0.640)	0.503	M	0.510(1.265)	0.001	M
1,3-diaminopropane	GC-MS	3.434(4.157)	0.892(6.502)	0.084(0.890)	0.008	M	0.087(0.041)	0.000	M
1,7-Dimethyluric acid	LC-MS (-)	2.733(8.592)	0.391(0.358)	0.169(0.169)	0.053	M	0.426(0.225)	0.417	M
10-hydroxydecanoic acid	GC-MS	0.481(1.048)	0.218(0.108)	0.175(0.167)	0.710	M	1.220(2.009)	0.000	M
12-deoxy-5beta-hydroxy-phorbol-13-hexadecanoate	LC-MS (+)	1.009(0.317)	1.100(0.258)	1.201(0.173)	0.084	M	1.070(0.278)	0.678	M
12-Desoxydiacetylpyxinol	LC-MS (+)	0.967(0.409)	0.866(0.381)	0.864(0.166)	0.385	T	1.064(0.423)	0.099	M
13S-hydroxyoctadecadienoic acid	LC-MS/MS (+)	0.895(0.344)	0.781(0.414)	0.899(0.375)	0.980	T	0.878(0.311)	0.990	M
16-Hydroxy hexadecanoic acid	LC-MS (-)	1.004(0.429)	1.107(0.612)	0.909(0.573)	0.030	M	1.020(0.787)	0.637	M
1-cyclohexyl-1-phenyl-3-piperidin-1-ylpropan-1-ol	LC-MS (+)	1.256(1.308)	0.956(1.638)	0.790(0.811)	1.000	M	0.513(0.884)	0.307	M
1-methyladenosine	GC-MS	0.427(1.207)	0.099(0.126)	0.105(0.131)	0.799	M	0.040(0.080)	0.094	M

Metabolite	Method	Patient group		Treatment group			Healthy Group		
		Mean	Median	Mean(SD) or Median(IQR)	P value	Test*	Mean(SD) or Median(IQR)	Pvalue	Test*
2-(7-methoxy-2-oxochromen-8-yl)-3-methylbut-2-enal	LC-MS (+)	1.001(0.367)	0.996(0.345)	0.955(0.253)	0.690	T	0.934(0.279)	0.371	M
2,3',4,6-Tetrahydroxybenzophenone	LC-MS (+)	0.434(0.661)	0.113(0.479)	0.323(0.750)	0.030	M	1.692(2.168)	0.013	M
2,3-dihydroxypropyl stearate/MG(18:0/0:0/0:0)	LC-MS/MS (+)	0.178(0.076)	0.172(0.066)	0.142(0.068)	0.149	M	0.184(0.124)	0.488	M
2,3-dimethylsuccinic acid	GC-MS	0.630(2.303)	0.015(0.024)	0.003(0.007)	0.036	M	0.036(0.583)	0.034	M
2-Amino-3-methylimidazo[4,5-f]quinoline	LC-MS (-)	3.306(12.150)	0.059(0.000)	0.059(0.000)	0.474	M	0.174(0.384)	0.005	M
2-aminoethanethiol	GC-MS	1.135(1.986)	0.421(0.482)	0.148(0.231)	0.009	M	1.508(1.211)	0.015	M
2-Arachidonylglycerol	LC-MS/MS (+)	0.820(0.400)	0.894(0.288)	0.859(0.320)	0.891	M	1.000(0.400)	0.176	T
2-butyne-1,4-diol	GC-MS	1.208(1.188)	0.763(1.259)	0.392(0.778)	0.138	M	0.794(0.451)	0.891	M
2-dec-9-enyl-3-hydroxypentanedioic acid	LC-MS/MS (+)	0.508(1.792)	0.017(0.000)	0.017(0.000)	0.666	M	0.074(0.197)	0.016	M
2-Furoic acid	LC-MS (-)	1.096(0.867)	0.817(0.683)	0.700(0.740)	0.190	M	0.887(0.770)	0.870	M
2-Hydroxyatrazine	LC-MS (+)	0.857(0.461)	0.789(0.574)	1.024(0.333)	0.269	T	1.036(0.458)	0.241	T
2-hydroxybutyric acid	GC-MS	1.586(1.257)	1.509(1.084)	1.273(1.938)	0.681	M	0.200(0.120)	0.000	M
2-hydroxyphenylacetic acid	GC-MS	0.924(1.386)	0.446(0.505)	0.383(0.489)	0.421	M	1.154(0.639)	0.010	M
2-Hydroxypropyl 2-isopropyl-5-methylcyclohexyl carbonate	LC-MS/MS (-)	0.457(0.000)	0.457(0.000)	0.457(0.000)	0.208	M	0.457(1.153)	0.007	M
2-isopropylmalic acid	GC-MS	0.925(1.203)	0.463(0.432)	0.205(0.201)	0.064	M	0.582(2.043)	0.206	M
2-ketoadipic acid	GC-MS	1.058(1.104)	0.647(1.119)	0.157(0.338)	0.003	M	0.994(1.582)	0.116	M
2-Ketobutyric acid	LC-MS (-)	0.583(0.463)	0.371(0.000)	0.371(0.000)	0.796	M	1.340(1.607)	0.009	M
2-ketocaproic acid	GC-MS	1.229(1.087)	0.675(1.312)	0.589(0.893)	0.356	M	0.887(0.624)	0.794	M
2-ketoisocaproic acid	GC-MS	0.922(1.055)	0.568(0.489)	0.563(0.320)	0.653	M	0.534(0.357)	0.525	M
2-keto-L-gulonic acid	GC-MS	3.845(5.379)	0.019(9.994)	0.003(0.007)	0.118	M	0.002(0.004)	0.094	M
2-methylanthra-9,10-quinone	LC-MS (+)	0.624(0.769)	0.373(0.425)	0.591(0.604)	0.377	M	0.843(1.151)	0.017	M
2-Octenoylcarnitine	LC-MS/MS (+)	0.667(1.249)	0.128(0.632)	0.098(0.164)	0.321	M	0.668(1.342)	0.051	M
2-piperidone	GC-MS	0.511(0.440)	0.364(0.497)	0.286(0.442)	0.891	M	1.398(0.589)	0.000	M
3-(Methylthio)propal	LC-MS/MS (+)	0.447(0.651)	0.177(0.225)	0.347(0.477)	0.279	M	0.971(2.190)	0.023	M

Metabolite	Method	Patient group		Treatment group			Healthy Group		
		Mean	Median	Mean(SD) or Median(IQR)	P value	Test*	Mean(SD) or Median(IQR)	Pvalue	Test*
3,3',4'5-Tetrahydroxystilbene	LC-MS (-)	0.647(0.652)	0.284(0.525)	0.284(0.784)	0.734	M	0.284(1.545)	0.336	M
3',4'-Dihydrodiol	LC-MS/MS (+)	0.589(1.683)	0.085(0.211)	0.138(0.148)	0.399	M	0.732(2.295)	0.012	M
3,4-dihydroxy-4-methylhexadecanoic acid	LC-MS/MS (+)	0.801(0.260)	0.762(0.367)	0.803(0.303)	0.681	M	0.843(0.230)	0.473	M
3,4-Dihydroxybenzeneacetic acid	LC-MS/MS (-)	0.592(0.759)	0.387(0.285)	0.398(0.346)	0.597	M	0.755(0.276)	0.000	M
3,4-dihydroxybenzoic acid	GC-MS	0.467(0.840)	0.233(0.162)	0.292(0.347)	0.597	M	1.234(1.723)	0.000	M
3,4-dihydroxyphenylacetic acid?	GC-MS	0.550(0.830)	0.266(0.394)	0.250(0.268)	0.769	M	1.421(1.154)	0.000	M
3,5-dimethoxy-4-hydroxycinnamic acid	GC-MS	0.631(1.754)	0.139(0.160)	0.102(0.074)	0.230	M	0.535(1.440)	0.000	M
3,7-dimethyl-6-octenyl acetate	LC-MS (+)	0.986(0.427)	1.096(0.675)	0.979(0.294)	0.960	T	1.077(0.432)	0.753	M
3-Aminoisobutanoic acid	LC-MS (+)	1.065(0.245)	1.136(0.064)	1.082(0.155)	0.922	M	1.006(0.155)	0.003	M
3-aminoisobutyric acid	GC-MS	0.742(1.090)	0.312(0.625)	0.225(0.279)	0.186	M	0.387(1.506)	0.362	M
3-aminopropionitrile	GC-MS	0.556(0.646)	0.337(0.623)	0.166(0.232)	0.200	M	1.447(1.039)	0.000	M
3-chloro-L-tyrosine	GC-MS	1.283(2.189)	0.404(0.924)	0.786(0.907)	0.653	M	0.416(0.629)	0.718	M
3-hexenedioic acid	GC-MS	3.120(4.848)	0.337(4.991)	0.132(0.352)	0.653	M	0.301(0.253)	0.700	M
3-Hydroxydodecanedioic acid	LC-MS/MS (-)	3.672(13.592)	0.033(0.000)	0.033(0.000)	0.495	M	0.087(0.321)	0.017	M
3-Hydroxyglutaric acid	LC-MS (-)	3.906(14.034)	0.156(0.106)	0.119(0.109)	0.302	M	0.172(0.102)	0.579	M
3-Hydroxymethylglutaric acid	LC-MS (-)	3.479(12.344)	0.085(0.172)	0.085(0.104)	0.905	M	0.085(0.454)	0.302	M
3-hydroxyphenylacetic acid	GC-MS	0.522(1.152)	0.172(0.228)	0.087(0.203)	0.830	M	0.852(1.594)	0.008	M
3-hydroxypropanoic acid	GC-MS	0.836(1.008)	0.587(0.528)	0.194(0.176)	0.010	M	1.130(1.193)	0.011	M
3-Hydroxysebacic acid	LC-MS (-)	3.296(11.976)	0.050(0.098)	0.050(0.000)	0.053	M	0.173(0.302)	0.146	M
3-indoleacetic acid	GC-MS	0.480(0.639)	0.208(0.454)	0.382(0.429)	0.316	M	1.483(1.071)	0.000	M
3-indolelactic acid	GC-MS	1.589(2.366)	0.646(1.321)	0.202(0.944)	0.118	M	0.502(0.846)	0.718	M
3-methyl-2-oxobutanoic acid	GC-MS	2.505(4.225)	0.502(0.477)	0.597(0.389)	0.799	M	0.559(0.382)	0.911	M
3-methyl-L-histidine	GC-MS	0.511(0.636)	0.270(0.363)	0.214(1.372)	0.860	M	0.130(0.364)	0.197	M
3-methyloxyindole	GC-MS	0.699(0.956)	0.378(0.999)	0.043(0.443)	0.246	M	0.130(3.298)	0.403	M
3-Methylthiopropionic acid	LC-MS/MS (+)	0.584(0.530)	0.435(0.106)	0.495(0.532)	0.830	M	1.043(1.361)	0.004	M

Metabolite	Method	Patient group		Treatment group			Healthy Group		
		Mean	Median	Mean(SD) or Median(IQR)	P value	Test*	Mean(SD) or Median(IQR)	Pvalue	Test*
3-Methylxanthine	LC-MS (-)	1.034(0.528)	1.146(0.859)	1.059(0.620)	0.904	T	0.904(0.986)	0.485	M
3-O-Methylglycyrol	LC-MS/MS (+)	0.306(0.458)	0.006(0.498)	0.006(0.478)	0.912	M	1.572(2.014)	0.000	M
3-phenyllactic acid	GC-MS	1.507(1.522)	1.044(1.675)	0.321(0.255)	0.021	M	0.721(0.772)	0.164	M
3-phosphoglycerate	GC-MS	0.471(0.459)	0.382(0.495)	0.195(0.485)	0.421	M	1.611(1.513)	0.000	M
4,8 Dimethylnonoyl carnitine	LC-MS/MS (+)	0.215(0.687)	0.031(0.000)	0.031(0.000)	0.474	M	0.402(2.057)	0.001	M
4-acetamidobutyric acid	GC-MS	1.682(1.373)	1.095(1.889)	0.691(0.710)	0.173	M	0.627(0.343)	0.006	M
4-Aminophenol	LC-MS/MS (+)	1.776(1.903)	0.744(1.913)	0.707(0.538)	0.356	M	0.650(0.765)	0.252	M
4-guanidinobutyric acid	GC-MS	0.235(0.243)	0.159(0.116)	0.163(0.116)	0.710	M	0.349(0.308)	0.007	M
4-hydroxy-3-methoxybenzoic acid	GC-MS	0.420(1.274)	0.065(0.072)	0.045(0.086)	0.468	M	0.252(3.506)	0.030	M
4-hydroxy-3-methoxybenzyl alcohol	GC-MS	0.191(0.545)	0.017(0.075)	0.096(0.092)	0.036	M	0.638(0.726)	0.000	M
4-hydroxy-3-methoxymandelic acid	GC-MS	0.553(0.575)	0.336(0.257)	0.206(0.193)	0.118	M	1.627(0.816)	0.000	M
4-hydroxy-4-(pyridin-2-yl)butan-2-one	LC-MS (+)	2.403(2.384)	1.590(3.845)	0.425(0.753)	0.064	M	0.453(0.226)	0.011	M
4-hydroxybenzoic acid	GC-MS	0.884(0.627)	0.749(0.632)	0.605(0.344)	0.152	T	1.277(1.103)	0.110	M
4-hydroxy-L-proline	GC-MS	2.110(2.011)	0.960(3.491)	0.363(0.996)	0.048	M	0.539(0.280)	0.039	M
4-hydroxymandelic acid	GC-MS	0.429(0.955)	0.163(0.105)	0.121(0.182)	0.493	M	0.851(1.155)	0.000	M
4-hydroxyphenylacetic acid	GC-MS	0.720(1.010)	0.138(1.130)	0.122(0.138)	0.444	M	1.140(1.868)	0.002	M
4-hydroxyphenylpyruvic acid	GC-MS	2.454(1.906)	3.584(3.956)	0.003(3.752)	0.149	M	0.045(0.056)	0.053	M
4-Methylumbelliferyl acetate	LC-MS (-)	0.391(1.015)	0.086(0.000)	0.086(0.000)	0.828	M	0.086(0.064)	0.503	M
5-Acetyl-2,4-dimethylthiazole	LC-MS/MS (+)	1.388(1.001)	1.169(1.875)	1.194(1.040)	0.984	M	0.111(0.686)	0.006	M
5-aminovaleric acid	GC-MS	0.017(0.016)	0.013(0.012)	1.101(3.762)	0.000	M	0.046(0.024)	0.000	M
5'-deoxy-5'-(methylthio)adenosine	GC-MS	1.053(0.155)	1.067(0.212)	1.005(0.157)	0.398	T	1.049(0.134)	0.718	M
5-hydroxyindole-3-acetic acid?	GC-MS	0.709(0.613)	0.462(1.008)	0.228(0.222)	0.092	M	1.304(0.540)	0.008	M
5-hydroxy-L-tryptophan	GC-MS	1.049(0.570)	1.116(0.617)	1.426(0.523)	0.068	T	0.494(0.787)	0.084	M
5-Hydroxymethyl tolterodine	LC-MS/MS (+)	0.331(0.872)	0.053(0.135)	0.070(0.111)	0.888	M	1.861(1.736)	0.000	M

Metabolite	Method	Patient group		Treatment group			Healthy Group		
		Mean	Median	Mean(SD) or Median(IQR)	P value	Test*	Mean(SD) or Median(IQR)	Pvalue	Test*
5-O-methylvisammioside	LC-MS (+)	1.136(0.289)	1.198(0.306)	0.979(0.200)	0.099	T	0.952(0.327)	0.069	T
6-deoxy-D-glucose	GC-MS	0.752(1.068)	0.356(0.815)	0.155(0.342)	0.138	M	1.822(2.493)	0.234	M
6-hydroxy caproic acid	GC-MS	0.617(0.567)	0.441(0.468)	0.250(0.194)	0.246	M	1.072(0.419)	0.003	M
6-hydroxybenzofuran-3(2H)-one	LC-MS (+)	0.335(1.180)	0.014(0.030)	0.015(0.065)	0.744	M	0.131(0.465)	0.004	M
6-hydroxyhexanoic acid	GC-MS	0.647(0.660)	0.408(0.636)	0.220(0.161)	0.036	M	1.114(0.797)	0.001	M
6-Keto-decanoylcarnitine	LC-MS/MS (+)	0.596(1.742)	0.088(0.174)	0.107(0.189)	0.677	M	0.983(1.302)	0.000	M
6-methoxy-7-(3-methylbut-2-enoxy)chromen-2-one	LC-MS/MS (+)	0.795(0.244)	0.722(0.224)	0.912(0.344)	0.280	T	0.981(0.726)	0.231	M
8-Deoxy-lactucin	LC-MS (+)	0.826(0.210)	0.847(0.218)	0.893(0.312)	0.444	M	1.004(0.593)	0.222	M
9-Decenoylcarnitine	LC-MS/MS (+)	0.546(1.806)	0.035(0.101)	0.038(0.082)	0.570	M	0.929(0.796)	0.000	M
9-methanesulfinylnonanoic acid	LC-MS (+)	1.031(0.170)	0.999(0.199)	1.083(0.164)	0.398	T	1.015(0.213)	0.623	M
Acetyl-N-formyl-5-methoxykynuremine	LC-MS/MS (+)	0.595(1.222)	0.174(0.474)	0.320(0.308)	0.493	M	0.951(1.713)	0.009	M
Aconitic acid	GC-MS	0.508(0.504)	0.317(0.410)	0.222(0.318)	0.246	M	1.560(0.804)	0.000	M
Adipamide	GC-MS	0.980(1.079)	0.609(0.960)	0.192(0.719)	0.128	M	0.893(1.020)	0.462	M
Adipic acid	GC-MS	0.637(0.408)	0.552(0.482)	0.303(0.317)	0.059	M	1.508(0.710)	0.000	T
Alanine	GC-MS	1.736(1.535)	1.004(2.430)	0.316(0.509)	0.009	M	0.558(0.525)	0.037	M
Allo-inositol	GC-MS	1.392(0.635)	1.374(0.689)	1.266(0.956)	0.665	T	0.702(0.287)	0.001	T
Alpha ketoglutaric acid	GC-MS	1.062(0.848)	0.763(1.436)	0.214(0.672)	0.053	M	1.121(0.657)	0.820	T
Alpha-Keto-gamma-(methylthio)butyric acid	LC-MS (+)	1.014(0.144)	1.044(0.230)	0.994(0.204)	0.570	M	0.918(0.303)	0.212	M
Alpha-N-Phenylacetyl-L-glutamine	LC-MS/MS (+)	0.567(1.286)	0.108(0.376)	0.234(0.166)	0.421	M	1.053(1.644)	0.012	M
Alpha-Tocopherol	LC-MS (+)	0.996(0.284)	1.078(0.218)	1.058(0.153)	0.891	M	1.110(0.184)	0.588	M
Altrose	GC-MS	0.435(0.657)	0.093(0.474)	0.207(0.143)	0.297	M	1.391(1.396)	0.000	M
Arachidic acid	GC-MS	1.006(0.240)	0.995(0.205)	0.939(0.191)	0.404	T	1.038(0.190)	0.661	T
Arbutin	GC-MS	0.394(0.347)	0.254(0.374)	0.548(0.736)	0.036	M	0.734(0.797)	0.004	M

Metabolite	Method	Patient group		Treatment group			Healthy Group		
		Mean	Median	Mean(SD) or Median(IQR)	P value	Test*	Mean(SD) or Median(IQR)	Pvalue	Test*
Arginyl-Valine	LC-MS/MS (+)	0.355(1.048)	0.020(0.148)	0.025(0.117)	0.873	M	1.064(2.256)	0.000	M
Ascorbic acid	GC-MS	1.061(1.420)	0.424(1.067)	0.152(0.226)	0.092	M	0.541(0.744)	0.794	M
Asparagine	GC-MS	2.083(2.840)	1.476(1.542)	0.273(1.077)	0.149	M	0.237(0.210)	0.028	M
Aspartic acid	GC-MS	0.878(1.655)	0.148(0.404)	0.063(0.096)	0.021	M	0.077(0.318)	0.071	M
Azelaic acid	LC-MS/MS (-)	0.061(0.043)	0.035(0.080)	0.058(0.100)	0.514	M	0.268(1.265)	0.011	M
B-Carotene	LC-MS (+)	1.028(0.260)	1.078(0.099)	1.130(0.064)	0.279	M	1.053(0.160)	0.284	M
Behenic acid	LC-MS (+)	0.958(0.361)	0.916(0.265)	0.897(0.096)	0.551	T	1.070(0.323)	0.099	M
Benzoic acid	GC-MS	0.375(0.321)	0.293(0.263)	0.260(0.268)	0.922	M	0.444(0.409)	0.053	M
Benzoylformic acid	GC-MS	1.417(3.836)	0.087(0.194)	0.210(1.857)	0.100	M	0.215(0.234)	0.032	M
Benzyl methyl sulfide	LC-MS (+)	0.838(0.464)	0.758(0.542)	1.362(0.687)	0.018	T	0.789(0.790)	0.930	M
Beta- alanine	GC-MS	0.610(0.618)	0.398(0.415)	0.138(0.433)	0.138	M	1.069(0.837)	0.003	M
Betaine	LC-MS/MS (+)	2.215(3.468)	1.010(1.906)	0.657(0.549)	0.173	M	0.134(0.262)	0.000	M
Beta-Sitosterol	LC-MS (+)	0.951(0.648)	0.830(0.950)	1.291(0.812)	0.205	T	0.762(1.400)	0.586	M
Biotin	LC-MS/MS (+)	0.454(0.827)	0.246(0.160)	0.293(0.461)	0.512	M	1.131(1.815)	0.010	M
C17 Sphinganine	LC-MS/MS (+)	1.042(0.968)	0.691(0.542)	0.531(0.932)	0.739	M	0.871(0.988)	0.659	M
Canavanine	GC-MS	2.809(3.688)	1.635(3.873)	0.087(1.070)	0.077	M	0.007(0.007)	0.000	M
Catechin	GC-MS	0.740(2.253)	0.126(0.063)	0.136(0.092)	0.860	M	0.229(0.710)	0.008	M
Catechol	GC-MS	0.371(0.236)	0.279(0.218)	0.388(0.254)	0.149	M	0.922(1.719)	0.000	M
Cellobiose	GC-MS	1.141(2.227)	0.261(1.130)	0.108(0.214)	0.084	M	1.114(1.489)	0.017	M
Chlorogenic acid	GC-MS	0.449(0.269)	0.381(0.234)	0.245(0.110)	0.013	M	0.407(0.386)	0.794	M
Cholesterol	GC-MS	1.455(1.724)	0.848(1.262)	0.504(0.572)	0.036	M	0.941(0.517)	0.832	M
Cholic acid	GC-MS	0.517(1.015)	0.238(0.231)	0.189(0.080)	0.316	M	0.235(0.223)	0.700	M
Cholic Acid, Methyl Ester	LC-MS (+)	0.906(0.274)	0.954(0.172)	1.116(0.263)	0.044	M	1.068(0.309)	0.110	M
Cinnamic acid	GC-MS	2.543(6.365)	0.063(0.243)	0.005(0.028)	0.109	M	0.004(0.010)	0.300	M

Metabolite	Method	Patient group		Treatment group			Healthy Group		
		Mean	Median	Mean(SD) or Median(IQR)	P value	Test*	Mean(SD) or Median(IQR)	Pvalue	Test*
Cis-4-hydroxycyclohexanecarboxylic acid	GC-MS	0.307(0.272)	0.309(0.239)	0.349(0.262)	0.597	M	0.708(0.605)	0.000	M
Citraconic acid	GC-MS	0.196(0.153)	0.168(0.171)	0.053(0.108)	0.029	M	0.692(0.904)	0.003	M
Citramalic acid	GC-MS	0.639(0.506)	0.489(0.557)	0.359(0.234)	0.073	T	1.547(0.777)	0.000	T
Citric acid	GC-MS	1.013(0.680)	0.970(1.157)	0.323(0.926)	0.059	M	1.262(0.528)	0.241	T
Citrulline	GC-MS	2.007(1.977)	0.945(3.489)	0.485(0.763)	0.100	M	0.485(0.325)	0.013	M
Cliosterol	LC-MS (+)	0.992(0.868)	0.789(0.522)	1.376(1.053)	0.112	M	0.672(1.021)	0.614	M
Creatinine	GC-MS	0.631(0.356)	0.572(0.316)	0.484(0.427)	0.149	M	1.472(0.606)	0.000	T
Cyclamic acid	LC-MS (-)	2.187(7.869)	0.026(0.032)	0.070(0.366)	0.209	M	0.168(0.875)	0.194	M
Cyclohexane-1,2-diol	GC-MS	0.698(0.747)	0.472(1.344)	0.041(1.353)	0.984	M	1.539(0.424)	0.004	M
Cystine	GC-MS	2.153(2.722)	0.801(2.396)	0.428(0.790)	0.064	M	0.353(0.482)	0.024	M
Cytidine	GC-MS	1.415(1.509)	0.970(1.919)	0.278(0.893)	0.173	M	1.150(0.459)	0.681	M
Cytosine	GC-MS	0.799(1.204)	0.389(0.607)	0.188(0.162)	0.059	M	1.251(1.150)	0.005	M
Dantron	LC-MS (-)	1.138(0.544)	1.158(0.759)	1.087(0.463)	0.784	T	1.026(0.746)	0.173	M
Decanoylcarnitine	LC-MS/MS (+)	0.402(1.339)	0.023(0.059)	0.005(0.141)	0.726	M	0.626(1.681)	0.000	M
Dehydroascorbic acid	GC-MS	1.032(1.181)	0.358(1.064)	0.190(0.595)	0.019	M	0.573(0.814)	0.681	M
Dehydrophytosphingosine	LC-MS (+)	1.143(0.807)	0.930(0.671)	0.560(0.558)	0.071	M	0.887(0.856)	0.890	M
Dicrotalic acid	GC-MS	0.373(0.424)	0.253(0.298)	0.509(1.566)	0.230	M	0.507(0.417)	0.018	M
Didanosine	LC-MS (+)	0.627(0.999)	0.331(0.506)	0.368(0.357)	0.751	M	0.789(0.928)	0.033	M
Dithiothreitol	GC-MS	0.892(0.768)	0.655(0.863)	0.262(0.350)	0.029	M	1.532(1.496)	0.084	M
Dopamine	GC-MS	0.712(1.158)	0.367(0.684)	0.196(0.262)	0.161	M	1.157(0.997)	0.000	M
Epsilon-caprolactam	GC-MS	0.357(0.673)	0.145(0.377)	0.310(0.280)	0.297	M	1.631(0.704)	0.000	M
Erucamide	LC-MS (+)	0.016(0.010)	0.014(0.012)	0.019(0.016)	0.029	M	0.018(0.013)	0.548	T
Estradiol	GC-MS	3.213(4.385)	0.579(6.596)	0.074(0.236)	0.026	M	0.325(0.231)	0.234	M
Estrone	LC-MS (-)	0.600(0.987)	0.172(0.173)	0.172(0.217)	0.867	M	1.003(1.418)	0.066	M
Fructose	GC-MS	0.420(0.334)	0.351(0.411)	0.296(0.373)	0.710	M	1.116(2.118)	0.003	M
Fucose	GC-MS	0.651(0.960)	0.309(0.474)	0.193(0.312)	0.279	M	1.486(0.826)	0.000	M

Metabolite	Method	Patient group		Treatment group			Healthy Group		
		Mean	Median	Mean(SD) or Median(IQR)	P value	Test*	Mean(SD) or Median(IQR)	Pvalue	Test*
Fumaric acid	GC-MS	2.464(2.345)	1.457(2.881)	0.318(0.347)	0.000	M	0.439(0.259)	0.000	M
Galactinol	GC-MS	1.089(0.756)	0.891(0.328)	0.791(0.213)	0.297	M	0.946(0.299)	0.417	M
Galactose	GC-MS	2.690(3.936)	0.942(3.280)	0.205(0.282)	0.064	M	0.054(0.065)	0.000	M
Galacturonic acid	GC-MS	0.804(0.734)	0.603(0.803)	0.398(0.451)	0.262	M	1.223(0.668)	0.017	M
Gelsenicine	LC-MS (+)	0.814(1.060)	0.544(0.785)	0.281(0.482)	0.356	M	1.148(0.967)	0.006	M
Gentisic acid	GC-MS	0.471(0.496)	0.347(0.223)	0.388(0.310)	0.891	M	1.500(1.461)	0.000	M
Glucoheptonic acid	GC-MS	0.992(1.037)	0.368(2.045)	0.214(0.384)	0.053	M	1.321(1.368)	0.071	M
Gluconic acid	GC-MS	2.393(3.805)	0.404(2.594)	0.109(0.997)	0.118	M	0.222(0.188)	0.094	M
Gluconic acid lactone	GC-MS	1.279(1.515)	0.518(2.181)	0.151(0.092)	0.161	M	0.507(0.659)	0.891	M
Glucosamine-phosphate	GC-MS	0.544(0.735)	0.226(0.636)	0.092(0.395)	0.544	M	0.466(0.860)	0.336	M
Glucosaminic acid	GC-MS	1.940(2.035)	1.021(3.435)	0.367(0.377)	0.044	M	0.715(0.758)	0.255	M
Glucose	GC-MS	1.513(1.933)	0.688(1.740)	0.766(2.058)	0.681	M	0.159(0.096)	0.007	M
Glucose-6-phosphate	GC-MS	1.283(1.573)	0.852(1.198)	0.421(0.652)	0.262	M	1.042(0.823)	0.375	M
Glucuronic acid	GC-MS	0.424(0.372)	0.271(0.191)	0.206(0.136)	0.279	M	1.725(0.880)	0.000	M
Glutamic acid	GC-MS	0.623(0.464)	0.501(0.519)	0.466(0.596)	0.356	M	1.210(1.540)	0.017	M
Glutamine	GC-MS	3.162(9.909)	0.084(0.537)	0.066(0.108)	0.468	M	0.175(0.169)	0.288	M
Glutamine (L)	LC-MS (+)	0.727(0.900)	0.215(1.201)	0.655(0.750)	0.560	M	1.458(1.451)	0.028	M
Glyceraldehyde	GC-MS	0.732(0.671)	0.613(0.567)	0.215(0.421)	0.064	M	0.762(2.031)	0.794	M
Glyceric acid	GC-MS	0.800(0.814)	0.462(0.706)	0.115(0.480)	0.053	M	0.128(0.345)	0.013	M
Glycerol	GC-MS	1.038(2.450)	0.144(0.728)	0.173(0.354)	0.984	M	0.246(0.880)	1.000	M
Glycerol 1-hexadecanoate	LC-MS (+)	0.948(0.270)	0.951(0.133)	1.126(0.187)	0.049	T	0.998(0.215)	0.488	M
Glycerol 1-octadecanoate	LC-MS (+)	1.016(0.332)	1.071(0.137)	1.136(0.270)	0.036	M	1.095(0.206)	0.696	M
Glycerol-phosphate	GC-MS	0.651(0.591)	0.421(0.675)	0.244(0.504)	0.316	M	1.119(0.634)	0.015	M
Glycine	GC-MS	1.870(1.681)	1.489(2.370)	0.901(0.950)	0.279	M	0.388(0.389)	0.002	M
Glycohyocholic acid	LC-MS (+)	0.243(0.830)	0.009(0.007)	0.009(0.032)	0.626	M	0.172(1.854)	0.049	M
Glycolic acid	GC-MS	0.512(0.396)	0.380(0.579)	0.270(0.430)	0.200	M	1.299(1.236)	0.000	M

Metabolite	Method	Patient group		Treatment group			Healthy Group		
		Mean	Median	Mean(SD) or Median(IQR)	P value	Test*	Mean(SD) or Median(IQR)	Pvalue	Test*
Glycyrrhetinic acid	LC-MS (+)	0.993(0.293)	1.094(0.487)	0.963(0.244)	0.760	T	0.905(0.316)	0.605	M
Gly-pro	GC-MS	0.569(1.072)	0.092(0.434)	0.089(0.071)	0.316	M	1.281(1.912)	0.002	M
Guanidine	LC-MS (+)	0.440(0.936)	0.226(0.165)	0.131(0.281)	0.550	M	1.369(1.629)	0.000	M
Guanidinosuccinic acid	GC-MS	0.383(0.823)	0.089(0.212)	0.171(0.534)	0.625	M	1.194(1.578)	0.000	M
Guanosine	LC-MS (+)	0.630(1.594)	0.031(0.410)	0.166(0.229)	0.447	M	1.164(1.766)	0.013	M
Heptadecanoic acid	GC-MS	0.651(0.469)	0.508(0.610)	0.281(0.654)	0.173	M	1.303(0.667)	0.000	M
Heptanoylcarnitine	LC-MS/MS (+)	0.348(0.951)	0.017(0.172)	0.012(0.071)	0.587	M	0.628(0.778)	0.000	M
Hippuric acid	GC-MS	0.490(0.600)	0.240(0.229)	0.436(0.587)	0.246	M	1.501(1.811)	0.000	M
Histidine	GC-MS	0.612(0.737)	0.233(1.010)	0.137(0.266)	0.149	M	0.408(1.818)	0.375	M
Homoserine	GC-MS	1.250(0.843)	0.981(1.437)	1.008(0.670)	0.392	T	0.909(0.960)	0.180	M
Hydroquinone	GC-MS	0.423(0.437)	0.364(0.390)	0.103(0.665)	0.681	M	1.688(0.515)	0.000	M
Hydroxyurea	GC-MS	1.220(1.208)	0.650(1.284)	0.373(0.755)	0.059	M	0.791(0.451)	0.737	M
Hyocholic acid	LC-MS (+)	1.068(0.102)	1.067(0.083)	1.076(0.104)	0.953	M	1.011(0.121)	0.094	M
Hypaconine	LC-MS (+)	2.258(7.349)	0.144(0.320)	0.110(0.242)	0.356	M	0.306(0.754)	0.399	M
Hypotaurine	GC-MS	0.051(0.039)	0.053(0.060)	1.064(2.089)	0.000	M	0.045(0.023)	0.652	T
Hypoxanthine	GC-MS	0.194(0.339)	0.033(0.266)	0.025(0.099)	0.493	M	1.318(1.971)	0.000	M
Icos-19-ene-1,2,4-triol	LC-MS (+)	1.249(0.363)	1.207(0.448)	0.920(0.370)	0.019	T	0.917(0.275)	0.021	M
Iminodiacetic acid?1	GC-MS	0.995(0.303)	1.003(0.279)	0.971(0.375)	0.845	T	1.050(0.235)	0.556	T
Indoleacetic acid	LC-MS/MS (+)	0.347(0.772)	0.027(0.000)	0.027(0.000)	0.708	M	0.646(1.065)	0.001	M
Indoline	LC-MS (+)	2.494(2.302)	1.576(3.530)	0.457(0.622)	0.005	M	0.374(0.334)	0.000	M
Indoxyl sulfate	LC-MS (-)	0.006(0.009)	0.002(0.000)	0.002(0.000)	0.840	M	0.002(0.016)	0.111	M
Inosine	GC-MS	2.824(4.009)	0.424(3.363)	0.273(0.168)	0.032	M	0.310(0.170)	0.018	M
Isocitric acid	GC-MS	2.389(3.950)	0.067(4.335)	0.049(0.111)	0.336	M	0.111(0.084)	0.417	M
Isoleucine	GC-MS	2.252(2.580)	1.439(2.011)	0.681(1.276)	0.161	M	0.304(0.169)	0.000	M
Isomaltose	GC-MS	1.269(2.026)	0.428(1.503)	0.111(0.448)	0.032	M	0.624(0.923)	0.509	M

Metabolite	Method	Patient group		Treatment group			Healthy Group		
		Mean	Median	Mean(SD) or Median(IQR)	P value	Test*	Mean(SD) or Median(IQR)	Pvalue	Test*
Isopalmitic acid	LC-MS (-)	3.331(10.937)	0.241(0.595)	0.257(0.200)	0.765	M	0.244(0.257)	0.694	M
Isopropyl beta-D-1-thiogalactopyranoside?	GC-MS	1.036(0.925)	0.730(1.894)	0.274(0.333)	0.071	M	1.267(1.265)	0.164	M
Isoxanthopterin	GC-MS	0.254(0.725)	0.043(0.075)	0.047(0.046)	0.316	M	1.896(2.066)	0.000	M
Itaconic acid	GC-MS	0.052(0.088)	0.018(0.049)	0.080(0.120)	0.186	M	0.124(1.043)	0.004	M
L-2-Amino-4-methylenepentanedioic acid	LC-MS/MS (+)	1.727(1.592)	1.119(1.278)	0.892(1.025)	0.336	M	0.423(0.242)	0.000	M
Lactamide	GC-MS	2.316(7.884)	0.207(0.139)	0.127(0.141)	0.544	M	0.453(0.240)	0.009	M
Lactic acid	GC-MS	1.684(2.349)	0.444(1.582)	0.390(0.728)	0.625	M	0.240(0.577)	0.156	M
Lactobionic acid	GC-MS	0.555(1.173)	0.163(0.192)	0.203(0.147)	0.984	M	1.225(1.742)	0.000	M
Lactose	GC-MS	1.206(2.318)	0.353(1.319)	0.149(0.230)	0.118	M	1.129(1.652)	0.050	M
Lactulose	GC-MS	0.499(0.645)	0.323(0.666)	0.403(0.575)	0.653	M	1.525(2.072)	0.017	M
Leucine	GC-MS	0.372(0.778)	0.171(0.234)	0.143(0.217)	0.891	M	0.178(0.465)	0.737	M
Leucrose	GC-MS	2.004(3.493)	1.083(1.058)	0.315(0.620)	0.010	M	0.753(0.548)	0.375	M
L-Glutamic acid	LC-MS (-)	1.769(2.679)	0.657(0.785)	0.438(0.476)	0.376	M	0.438(0.000)	0.120	M
L-Glutamine	LC-MS (-)	0.124(0.129)	0.048(0.101)	0.127(0.227)	0.216	M	0.287(1.407)	0.018	M
L-Hexahydro-3-imino-1,2,4-oxadiazepine-3-carboxylic acid	LC-MS/MS (+)	1.676(1.449)	1.332(1.107)	0.914(0.929)	0.544	M	0.370(0.179)	0.001	M
L-Histidine	LC-MS/MS (-)	0.906(0.940)	0.816(1.046)	1.068(1.428)	0.435	M	0.479(0.942)	0.497	M
L-Homoserine	LC-MS (-)	4.674(17.236)	0.033(0.073)	0.013(0.017)	0.047	M	0.005(0.000)	0.000	M
Linoleic acid	LC-MS (-)	4.389(11.728)	0.067(0.075)	0.027(0.104)	0.646	M	0.029(0.056)	0.166	M
L-Octanoylcarnitine	LC-MS/MS (+)	0.602(1.767)	0.123(0.168)	0.058(0.122)	0.513	M	0.774(1.271)	0.001	M
L-Phenylalanine	LC-MS/MS (+)	0.761(2.274)	0.130(0.139)	0.080(0.123)	0.279	M	0.134(0.878)	0.811	M
L-Tryptophan	LC-MS/MS (+)	1.936(1.900)	0.938(2.755)	0.526(0.842)	0.036	M	0.621(0.664)	0.023	M
Lysine	GC-MS	3.510(4.399)	1.040(5.316)	0.098(0.618)	0.006	M	0.010(0.017)	0.000	M
Lysopa(18:0e/0:0)	LC-MS/MS (+)	0.561(0.809)	0.364(0.739)	0.082(0.185)	0.252	M	0.066(0.450)	0.326	M

Metabolite	Method	Patient group		Treatment group			Healthy Group		
		Mean	Median	Mean(SD) or Median(IQR)	P value	Test*	Mean(SD) or Median(IQR)	Pvalue	Test*
Lyxose	GC-MS	1.056(1.901)	0.092(0.394)	0.066(0.099)	0.297	M	0.478(0.752)	0.018	M
Maleamic acid	GC-MS	0.645(0.547)	0.521(0.372)	0.335(0.348)	0.161	M	1.464(0.645)	0.000	M
Maleic acid	GC-MS	0.807(1.164)	0.530(0.626)	0.224(0.987)	0.710	M	0.546(1.536)	0.403	M
Malic acid	GC-MS	2.597(2.684)	1.509(3.609)	0.284(0.364)	0.010	M	0.202(0.165)	0.000	M
Malonamide	GC-MS	0.428(0.166)	0.416(0.243)	0.296(0.139)	0.186	M	0.307(0.095)	0.064	M
Malonic acid	GC-MS	1.210(1.066)	0.771(1.685)	0.534(0.749)	0.200	M	0.951(0.588)	0.813	M
Maltitol	GC-MS	1.244(1.129)	0.685(0.464)	0.634(0.178)	0.262	M	0.613(0.340)	0.188	M
Maltose	GC-MS	2.088(4.434)	0.570(1.499)	0.092(0.299)	0.003	M	0.449(1.059)	0.645	M
Maltotriitol	GC-MS	1.026(1.159)	0.269(1.812)	0.115(0.782)	0.200	M	0.642(1.507)	0.389	M
Maltotriose	GC-MS	1.391(2.892)	0.161(0.335)	0.122(0.079)	0.215	M	0.244(0.717)	0.737	M
Mannitol	GC-MS	2.971(4.591)	0.221(6.167)	0.126(0.195)	0.048	M	0.323(0.457)	0.493	M
Mannose	GC-MS	1.109(1.009)	1.357(1.309)	0.187(0.428)	0.246	M	1.061(0.887)	0.871	M
Melezitose	GC-MS	2.769(9.395)	0.034(0.373)	0.015(0.042)	0.262	M	0.183(0.807)	0.080	M
Melibiose	GC-MS	1.693(2.982)	0.170(0.812)	0.073(0.105)	0.084	M	0.595(2.164)	0.105	M
Methionine	GC-MS	2.234(3.340)	0.573(2.855)	0.475(0.955)	0.518	M	0.480(0.400)	0.245	M
Methyl 15-hydroperoxy-9Z,12Z,16E-octadecatrienoate	LC-MS/MS (+)	0.797(0.342)	0.679(0.406)	0.829(0.414)	0.816	T	0.847(0.509)	0.554	M
Methyl 3-mercaptobutanoate	LC-MS/MS (+)	0.287(0.394)	0.125(0.000)	0.125(0.000)	0.577	M	0.125(0.000)	0.881	M
Methyl Stearate	GC-MS	1.050(0.363)	1.021(0.376)	1.011(0.257)	0.732	T	0.978(0.131)	0.756	M
Methylmalonic acid	GC-MS	1.461(1.521)	0.820(1.237)	0.323(0.612)	0.053	M	0.737(0.655)	0.493	M
MG(16:0/0:0/0:0)	LC-MS/MS (+)	0.443(0.175)	0.516(0.229)	0.422(0.232)	0.799	M	0.407(0.194)	0.554	M
MG(18:2(9Z,12Z)/0:0/0:0)	LC-MS (+)	1.041(0.100)	1.036(0.074)	1.056(0.082)	0.468	M	1.041(0.084)	0.910	M
MG(18:3(6Z,9Z,12Z)/0:0/0:0)	LC-MS/MS (+)	0.692(0.333)	0.724(0.469)	0.579(0.450)	1.000	M	0.662(0.381)	0.811	M
Mimosine	GC-MS	1.092(0.882)	0.755(1.211)	0.479(0.357)	0.200	M	0.849(0.353)	0.718	M
Monolinolein/MG(18:2(9Z,12Z)/0:0/0:0)	LC-MS/MS (+)	0.635(0.393)	0.618(0.376)	0.509(0.441)	0.922	M	0.434(0.285)	0.252	M

Metabolite	Method	Patient group		Treatment group			Healthy Group		
		Mean	Median	Mean(SD) or Median(IQR)	P value	Test*	Mean(SD) or Median(IQR)	Pvalue	Test*
Monoolein / MG(18:1(9Z)/0:0/0:0)	LC-MS/MS (+)	1.034(0.111)	1.032(0.130)	1.071(0.096)	0.335	T	1.058(0.106)	0.521	M
Mucic acid	GC-MS	0.975(1.065)	0.683(1.185)	0.227(0.286)	0.032	M	1.061(0.990)	0.094	M
Mycosporine serinol	LC-MS (+)	1.021(0.294)	1.080(0.084)	1.129(0.115)	0.118	M	1.037(0.101)	0.140	M
Myo-inositol	GC-MS	0.390(0.331)	0.315(0.272)	0.160(0.231)	0.084	M	0.416(0.316)	0.794	M
Myristic acid	GC-MS	0.936(0.268)	0.901(0.420)	0.931(0.231)	0.953	T	1.078(0.189)	0.090	T
N-(2-hydroxyethyl)iminodiacetic acid	GC-MS	0.641(0.550)	0.406(0.775)	0.338(0.527)	0.625	M	1.286(0.922)	0.004	M
N,N-(2,2-dihydroxy-ethyl) arachidonoyl amine	LC-MS/MS (+)	0.960(0.600)	0.901(0.771)	0.703(0.485)	0.399	M	0.828(0.469)	0.504	M
N,N-Dimethyldodecylamine N-oxide	LC-MS (+)	1.377(1.010)	1.301(1.223)	0.684(0.329)	0.036	M	0.915(0.659)	0.320	M
N,O-Didesmethylvenlafaxine	LC-MS/MS (+)	0.967(0.364)	0.933(0.491)	1.112(0.441)	0.324	T	0.924(0.388)	0.930	M
N-Acetylaminooctanoic acid	LC-MS/MS (+)	0.432(0.510)	0.322(0.346)	0.208(0.395)	0.891	M	1.374(1.383)	0.001	M
N-acetyl-D-glucosamine	GC-MS	0.575(0.442)	0.440(0.494)	0.328(0.216)	0.072	T	1.422(0.660)	0.000	M
N-acetyl-D-mannosamine	GC-MS	0.515(0.495)	0.325(0.519)	0.180(0.197)	0.186	M	1.643(0.989)	0.000	M
N-acetyl-D-tryptophan	GC-MS	2.685(6.619)	0.920(1.228)	0.332(0.693)	0.149	M	0.277(0.108)	0.000	M
N-acetyl-L-aspartic acid	GC-MS	0.822(0.695)	0.613(0.932)	0.166(0.330)	0.118	M	1.369(0.658)	0.021	T
N-acetyl-L-glutamic acid	GC-MS	0.525(0.395)	0.524(0.482)	0.302(0.613)	0.799	M	1.368(0.544)	0.000	T
N-acetyl-ornithine	GC-MS	0.569(0.421)	0.568(0.725)	0.164(0.711)	0.468	M	0.470(1.493)	0.756	M
NALPHA-ACETYL-L-LYSINE	LC-MS (+)	1.031(0.245)	1.074(0.108)	1.087(0.211)	0.421	M	1.007(0.156)	0.060	M
Naringin	GC-MS	1.850(5.354)	0.293(0.439)	0.215(0.127)	0.149	M	0.406(0.591)	0.180	M
N-carbamyl-L-glutamic acid 5	GC-MS	2.674(3.835)	0.552(2.972)	0.289(0.417)	0.064	M	0.330(0.527)	0.028	M
N-ethylglycine	GC-MS	1.753(2.030)	0.748(2.283)	0.493(0.725)	0.149	M	0.477(0.991)	0.094	M
N-isopropyl alpha-methylarachidonoyl amine	LC-MS/MS (+)	0.798(1.257)	0.465(0.080)	0.477(0.064)	0.953	M	0.471(0.087)	0.850	M
Nonoylcarnitine	LC-MS/MS (+)	0.323(0.971)	0.043(0.097)	0.038(0.078)	0.710	M	0.837(2.482)	0.000	M
Norepinephrine	GC-MS	2.302(3.572)	0.492(2.749)	0.221(0.180)	0.297	M	0.291(0.462)	0.245	M

Metabolite	Method	Patient group		Treatment group			Healthy Group		
		Mean	Median	Mean(SD) or Median(IQR)	P value	Test*	Mean(SD) or Median(IQR)	Pvalue	Test*
Norleucine	GC-MS	2.522(2.702)	1.313(2.925)	0.702(1.544)	0.297	M	0.223(0.139)	0.001	M
Norvaline	GC-MS	2.704(2.846)	1.519(3.965)	0.832(1.288)	0.138	M	0.153(0.109)	0.000	M
N-palmitoyl glutamine	LC-MS/MS (+)	1.031(0.582)	0.888(0.750)	0.774(0.560)	0.710	M	0.823(0.753)	0.641	M
N-tert-butyl arachidonoyl amine	LC-MS/MS (+)	0.051(0.039)	0.046(0.026)	0.050(0.026)	0.279	M	0.052(0.022)	0.529	M
Oleanonic acid	LC-MS/MS (+)	2.073(5.931)	0.366(0.901)	0.113(0.330)	0.388	M	0.173(0.579)	0.481	M
Oleic acid	GC-MS	0.565(0.486)	0.469(0.772)	0.212(0.427)	0.262	M	1.632(0.887)	0.000	T
O-phosphocolamine	GC-MS	0.608(0.547)	0.459(0.603)	0.232(0.478)	0.297	M	1.111(1.627)	0.000	M
Ornithine	GC-MS	2.994(4.223)	1.009(2.142)	0.283(0.639)	0.010	M	0.317(0.154)	0.001	M
Orotic acid	GC-MS	0.422(0.431)	0.254(0.358)	0.238(0.484)	0.769	M	1.229(0.648)	0.000	M
Oxalacetic acid	LC-MS/MS (-)	0.724(0.819)	0.284(0.255)	0.284(1.868)	0.824	M	2.013(1.930)	0.105	M
Oxalic acid	GC-MS	0.740(0.431)	0.814(0.501)	0.858(0.244)	0.597	M	1.091(0.576)	0.031	T
PAF (Platelet Activating Factor)	LC-MS (+)	1.009(0.306)	1.046(0.237)	1.073(0.211)	0.597	M	1.040(0.300)	0.990	M
Palatinitol	GC-MS	1.038(0.356)	0.962(0.491)	1.288(0.474)	0.104	T	0.826(0.291)	0.047	M
Palmitic acid	GC-MS	0.994(0.230)	0.992(0.238)	0.985(0.187)	0.903	T	1.017(0.168)	0.743	T
Palmitic amide	LC-MS/MS (+)	0.198(0.304)	0.065(0.000)	0.065(0.000)	0.957	M	0.065(0.000)	0.566	M
Palmitoleic acid	LC-MS (-)	0.087(0.081)	0.059(0.057)	0.039(0.029)	0.145	M	0.064(0.122)	0.889	M
P-Aminobenzoic acid	LC-MS (+)	0.981(0.390)	1.016(0.363)	1.201(0.347)	0.113	T	1.089(0.409)	0.588	M
Pantothenic acid	GC-MS	1.241(1.792)	0.425(1.584)	0.133(0.380)	0.092	M	1.309(1.116)	0.050	M
P-cresol	GC-MS	0.521(0.759)	0.201(0.408)	0.532(0.357)	0.040	M	1.021(1.481)	0.001	M
Pentadecanoic acid	LC-MS (-)	3.527(13.010)	0.055(0.019)	0.048(0.037)	0.675	M	0.061(0.068)	0.532	M
Phenylalanine	GC-MS	2.596(2.536)	1.275(3.929)	0.675(0.869)	0.048	M	0.198(0.200)	0.000	M
Phenyl-beta-glucopyranoside	GC-MS	2.738(3.607)	1.071(2.928)	0.602(1.115)	0.161	M	0.183(0.148)	0.000	M
Phenylethylamide	LC-MS/MS (+)	0.086(0.045)	0.072(0.030)	0.069(0.025)	0.799	M	0.070(0.025)	0.753	M
Phenylethylamine	GC-MS	0.012(0.013)	0.010(0.007)	1.293(4.590)	0.000	M	0.019(0.010)	0.001	M

Metabolite	Method	Patient group		Treatment group			Healthy Group		
		Mean	Median	Mean(SD) or Median(IQR)	P value	Test*	Mean(SD) or Median(IQR)	Pvalue	Test*
Phosphodimethylethanolamine	LC-MS/MS (+)	0.321(0.437)	0.177(0.392)	0.118(0.303)	0.642	M	1.254(1.525)	0.011	M
Phosphoric acid	GC-MS	1.041(1.359)	0.506(0.695)	0.363(0.263)	0.468	M	0.492(0.633)	0.775	M
Phthalic acid	GC-MS	0.496(1.039)	0.086(0.202)	0.071(0.066)	0.399	M	1.379(1.152)	0.000	M
P-hydroxyphenyllactic acid?	GC-MS	1.535(1.163)	0.966(1.460)	0.542(0.512)	0.017	M	0.702(0.297)	0.028	M
Phytosphingosine	LC-MS (+)	0.954(0.368)	0.904(0.380)	0.776(0.325)	0.279	M	1.139(0.687)	0.054	M
Picolonic acid	GC-MS	0.906(0.517)	0.773(0.289)	0.796(0.281)	0.860	M	1.249(0.349)	0.001	M
Pimelic acid	GC-MS	0.589(0.705)	0.403(0.144)	0.298(0.166)	0.149	M	1.307(1.094)	0.000	M
Porphine	GC-MS	0.695(0.294)	0.583(0.232)	0.692(0.215)	0.262	M	0.908(0.161)	0.000	M
Progesterone	LC-MS (+)	1.039(0.255)	1.085(0.096)	1.115(0.218)	0.399	M	0.991(0.133)	0.023	M
Proline	GC-MS	3.718(5.390)	1.003(4.419)	0.163(0.610)	0.008	M	0.013(0.012)	0.000	M
Proline betaine	LC-MS/MS (+)	0.687(2.462)	0.006(0.020)	0.014(0.059)	0.077	M	0.040(0.831)	0.005	M
Prunetin	GC-MS	1.077(0.180)	1.058(0.293)	0.974(0.174)	0.117	T	0.996(0.117)	0.139	T
Purine riboside	GC-MS	0.611(1.545)	0.143(0.335)	0.048(0.059)	0.059	M	0.081(2.504)	0.610	M
Pyridoxine	LC-MS/MS (+)	0.348(0.547)	0.218(0.267)	0.172(0.214)	0.677	M	0.873(2.121)	0.033	M
Pyrogallol	GC-MS	0.934(0.258)	0.885(0.323)	0.863(0.213)	0.415	T	1.120(0.156)	0.023	T
Pyroglutamic acid	GC-MS	1.564(1.056)	1.113(1.718)	0.569(0.472)	0.005	M	0.906(0.417)	0.040	T
Pyruvic acid	GC-MS	1.592(1.144)	1.228(1.721)	0.772(0.752)	0.279	M	0.577(0.230)	0.006	T
Quinic acid	GC-MS	0.681(0.857)	0.194(0.838)	0.262(0.224)	0.953	M	0.509(1.055)	0.197	M
Quinolinic acid	GC-MS	0.777(0.625)	0.620(0.542)	0.275(0.278)	0.029	M	1.366(1.226)	0.003	M
Raffinose	GC-MS	0.658(1.161)	0.188(0.443)	0.070(0.256)	0.149	M	1.027(2.034)	0.005	M
Ribitol	GC-MS	0.480(0.365)	0.447(0.314)	0.311(0.262)	0.246	M	1.574(0.692)	0.000	M
Ribonic acid-gamma-lactone	GC-MS	1.054(1.894)	0.443(0.662)	0.247(0.252)	0.048	M	1.298(0.463)	0.001	M
Ribose	GC-MS	0.587(0.510)	0.436(0.410)	0.290(0.278)	0.149	M	1.602(0.830)	0.000	M
Saccharic acid	GC-MS	0.677(0.480)	0.532(0.192)	0.501(0.595)	0.597	M	1.323(0.761)	0.000	M
Sedoheptulose	GC-MS	3.174(4.444)	0.987(3.503)	0.775(0.730)	0.064	M	0.031(0.038)	0.000	M

Metabolite	Method	Patient group		Treatment group			Healthy Group		
		Mean	Median	Mean(SD) or Median(IQR)	P value	Test*	Mean(SD) or Median(IQR)	Pvalue	Test*
Serine	GC-MS	1.483(1.672)	0.878(1.595)	0.312(0.348)	0.077	M	0.396(1.317)	0.311	M
Shikimic acid	GC-MS	2.045(2.566)	0.604(3.184)	0.139(0.202)	0.161	M	0.406(0.904)	0.852	M
Sophorose	GC-MS	1.168(2.206)	0.347(1.067)	0.236(0.305)	0.186	M	1.161(1.276)	0.034	M
Sorbitol	GC-MS	0.530(0.351)	0.416(0.375)	0.460(0.521)	0.953	M	0.799(0.969)	0.001	M
Spermine	LC-MS (-)	4.694(17.549)	0.001(0.003)	0.001(0.000)	0.060	M	0.001(0.000)	0.029	M
Squalene	GC-MS	1.316(1.593)	0.469(1.068)	0.439(0.348)	0.279	M	0.939(1.187)	0.245	M
Stearic acid	GC-MS	1.031(0.217)	1.023(0.158)	0.987(0.189)	0.555	T	1.001(0.157)	0.653	T
Suberic acid	LC-MS (-)	3.709(13.361)	0.085(0.088)	0.085(0.000)	0.054	M	0.143(0.270)	0.296	M
Succinic acid	GC-MS	0.458(0.483)	0.273(0.476)	0.094(0.152)	0.077	M	0.726(0.814)	0.013	M
Sucrose	GC-MS	0.577(0.955)	0.273(0.530)	0.176(0.358)	0.544	M	1.058(2.150)	0.075	M
Tagatose	GC-MS	0.926(0.879)	0.679(0.923)	0.196(0.306)	0.021	M	1.107(1.081)	0.277	M
Talose	GC-MS	1.997(1.011)	2.080(1.314)	2.691(2.120)	0.799	M	0.023(0.044)	0.000	M
Tartaric acid	GC-MS	0.247(0.687)	0.017(0.023)	0.617(2.203)	0.000	M	0.078(0.254)	0.001	M
Tartronic acid	GC-MS	0.539(0.454)	0.396(0.460)	0.191(0.320)	0.036	M	1.103(1.343)	0.001	M
Taurine	LC-MS (-)	0.394(0.199)	0.341(0.227)	0.777(1.212)	0.225	M	0.659(0.377)	0.002	M
Tetradecane	LC-MS (+)	1.003(0.332)	1.112(0.257)	1.067(0.178)	0.830	M	1.084(0.265)	0.850	M
Thiacremonone	LC-MS/MS (-)	2.809(9.357)	0.147(0.352)	0.053(0.322)	0.481	M	0.530(0.613)	0.023	M
Threitol	GC-MS	0.554(0.531)	0.431(0.433)	0.356(0.382)	0.399	M	1.541(1.369)	0.000	M
Threo-3-hydroxy-L-aspartate	GC-MS	0.426(0.413)	0.259(0.292)	0.269(0.249)	0.891	M	0.825(1.385)	0.010	M
Threo-beta-hydroxyaspartic acid?	GC-MS	2.495(2.533)	1.189(3.808)	0.470(0.927)	0.053	M	0.286(0.281)	0.000	M
Threonic acid	LC-MS (-)	0.829(0.875)	0.557(0.876)	0.468(0.500)	0.984	M	1.075(0.569)	0.045	M
Threonine	GC-MS	1.853(1.936)	1.039(2.302)	0.383(0.450)	0.084	M	0.185(0.230)	0.002	M
Threose	GC-MS	0.609(0.617)	0.399(0.415)	0.138(0.433)	0.149	M	1.073(0.837)	0.003	M
Thymidine	GC-MS	1.073(0.527)	1.143(0.664)	1.425(0.533)	0.077	T	0.465(0.878)	0.039	M
Thymine	GC-MS	1.008(1.228)	0.452(1.335)	0.219(0.627)	0.297	M	1.308(0.757)	0.044	M
Tiglylcarnitine	LC-MS/MS (-)	0.555(0.339)	0.475(0.308)	0.508(0.672)	0.830	M	0.472(0.434)	0.871	M

Metabolite	Method	Patient group		Treatment group			Healthy Group		
		Mean	Median	Mean(SD) or Median(IQR)	P value	Test*	Mean(SD) or Median(IQR)	Pvalue	Test*
Trimethyllysine	GC-MS	1.607(1.884)	0.803(1.747)	0.417(0.558)	0.138	M	0.487(0.553)	0.037	M
Tryptophan	GC-MS	2.729(3.883)	0.589(3.387)	0.193(0.423)	0.010	M	0.370(0.579)	0.075	M
Tyrosine	GC-MS	1.658(2.117)	0.892(1.687)	0.413(0.734)	0.084	M	0.651(0.721)	0.610	M
Uracil	GC-MS	0.335(0.393)	0.119(0.441)	0.179(0.266)	0.984	M	1.536(1.144)	0.000	M
Urea	GC-MS	1.840(4.316)	0.028(0.138)	0.034(0.395)	0.739	M	0.004(0.058)	0.255	M
Uric acid	GC-MS	1.048(0.689)	1.038(0.762)	0.369(0.574)	0.012	M	1.386(0.732)	0.206	M
Urocanic acid	GC-MS	0.397(0.918)	0.106(0.170)	0.097(0.027)	0.597	M	1.944(1.889)	0.000	M
Uurethane	GC-MS	1.453(1.581)	0.783(0.933)	0.302(0.766)	0.077	M	0.767(1.519)	0.255	M
Valine	GC-MS	2.322(2.446)	1.680(2.269)	0.667(1.845)	0.138	M	0.229(0.108)	0.000	M
Vitamin K1	LC-MS (+)	0.900(0.281)	0.931(0.159)	0.837(0.135)	0.149	M	1.020(0.284)	0.178	M
Xanthine	GC-MS	0.153(0.502)	0.003(0.012)	0.003(0.003)	0.922	M	1.590(1.562)	0.000	M
Xanthurenic acid	GC-MS	0.474(0.766)	0.204(0.319)	0.135(0.160)	0.356	M	1.412(1.446)	0.000	M
Xylitol	GC-MS	0.591(0.686)	0.383(0.516)	0.340(0.504)	0.518	M	1.587(0.697)	0.000	M

T: The groups which follow normal distribution were compared by two-tailed Student's t-test. M: Nonnormally distributed groups were carried out by Mann Whitney U test. SD: Standard deviation. IQR: Interquartile Range

Table S1.5. Statistical analysis results of metabolites involved in sulfur metabolism identified by LC-MS/MS from urine samples

Metabolite	Patient	Treatment		Healthy	
	Mean* ± SE	Mean* ± SE	p	Mean* ± SE	p
**Cystine**	14.776 ± 2.790	6.164 ± 1.367	0.007	1.124 ± 0.199	0.000
**Cysteine**	4.036 ± 0.107	3.976 ± 0.056	0.606	4.350 ± 0.115	0.094
**Serine**	25.995 ± 5.405	16.665 ± 2.938	0.131	9.782 ± 1.511	0.002
**Methionine**	0.452 ± 0.082	0.338 ± 0.058	0.251	0.247 ± 0.020	0.002
**Cysteamine**	0.075 ± 0.007	0.075 ± 0.006	0.998	0.086 ± 0.016	0.638
**Glutathione**	0.054 ± 0.004	0.050 ± 0.002	0.295	0.072 ± 0.006	0.047
**Hypotaurine**	0.188 ± 0.071	4.669 ± 1.569	0.032	1.515 ± 0.813	0.335

* All results are in ppm units.

PCA scoring graphic using all data obtained from urine metabolomics analysis showed that urine metabolic profiles were able to differentiate newly diagnosed cystinosis patient without treatment, cystinosis patients under treatment and healthy individuals **(Figure S1.5)**. PLS-DA graphs were obtained to evaluate differences between groups, VIP graphs showing most important metabolites leading differences in data analysis, R^2 and Q^2 values to determine confidence of PLS-DA and coefficient graphs to determine increasing and decreasing metabolites between groups. We saw that metabolomics profiles were completely different between study group that differentiate each other **(Figure S1.6-8)**. The fact that the obtained R^2 and Q^2 values are greater than 0.5 indicates the validity of the methods.

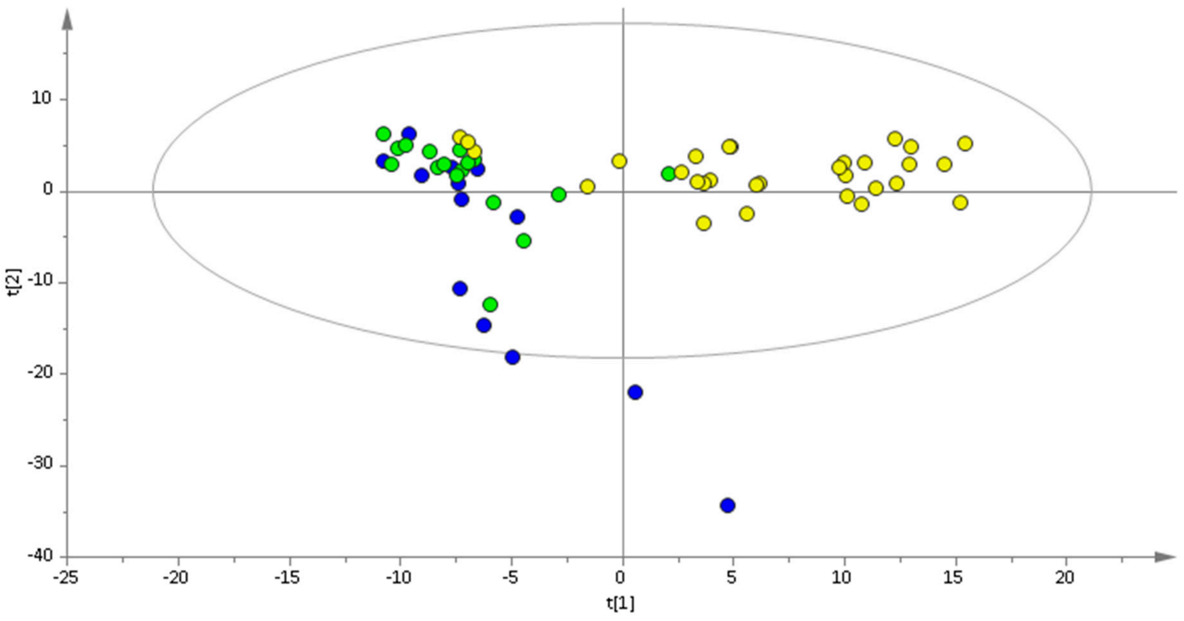

Figure S1.5. PCA graph of metabolomics analyses of urine samples through GC-MS, LC-qTOF-MS and LC-MS/MS (yellow dots: healthy; blue dots: patient group; green dots: treatment)

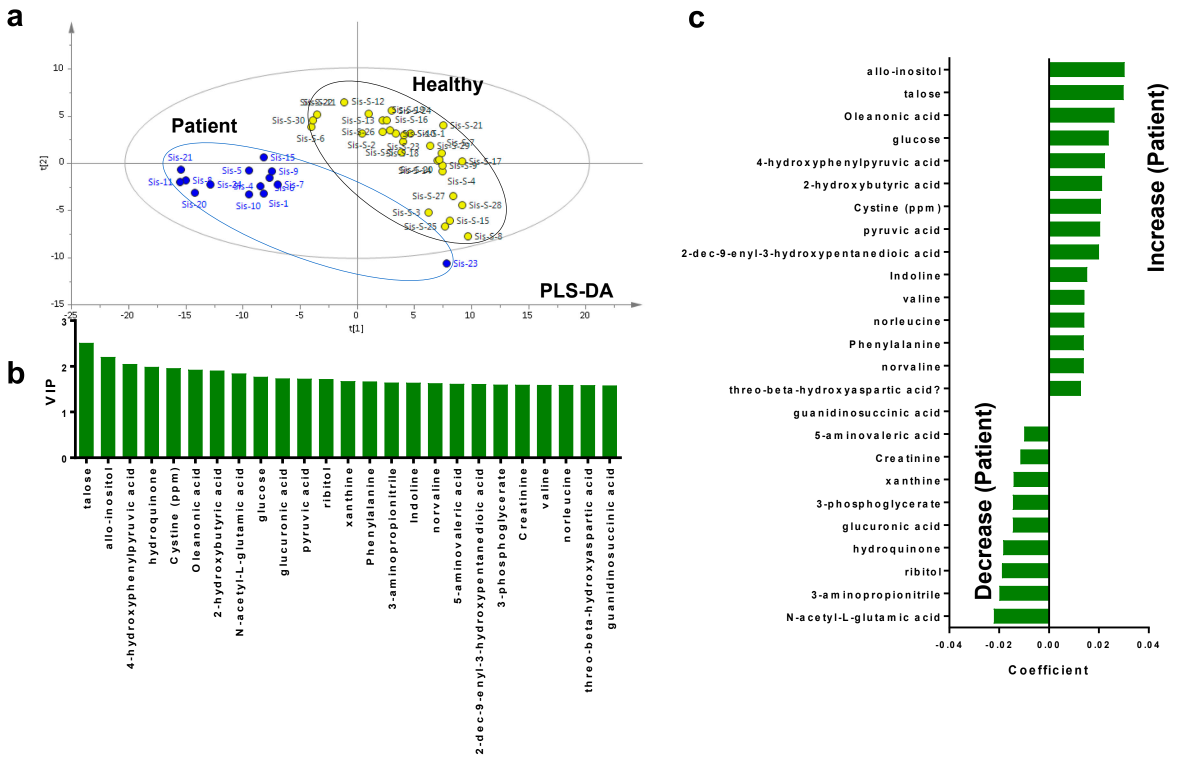

Figure S1.6. Multivariate analyses of data obtained from urine metabolomics analysis by GC-MS, LC-qTOF-MS and LC-MS/MS (patient group vs healthy group). **a)** PLS-DA score graph,

(R2: 0.855, Q2: 0.601) **b)** VIP graph showing most important metabolites leading differences in PLS-DA analysis **c)** Coefficient graph showing increasing and decreasing metabolites between groups

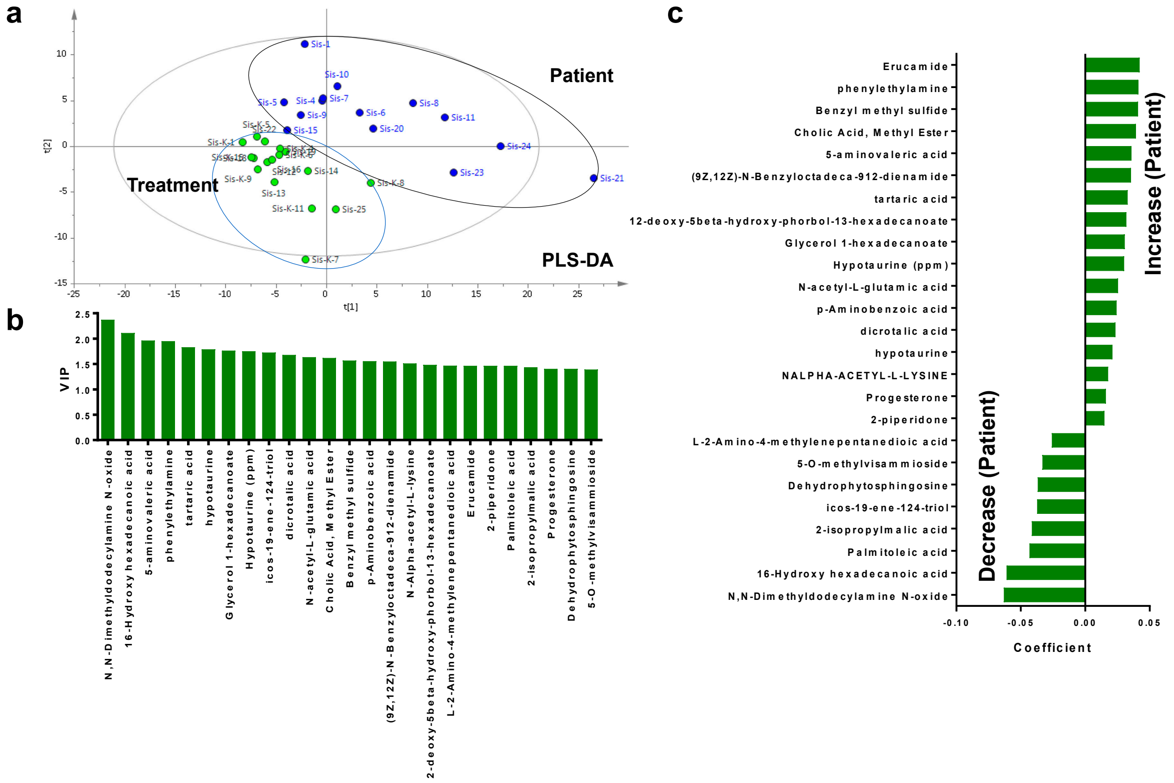

Figure S1.7. Multivariate analyses of data obtained from urine metabolomics analysis by GC-MS, LC-qTOF-MS and LC-MS/MS (patient group vs treatment groups). **a)** PLS-DA score graph (R^2: 0.941, Q^2: 0.561), **b)** VIP graph showing most important metabolites leading differences in data analysis **c)** Coefficient graph showing increasing and decreasing metabolites between groups

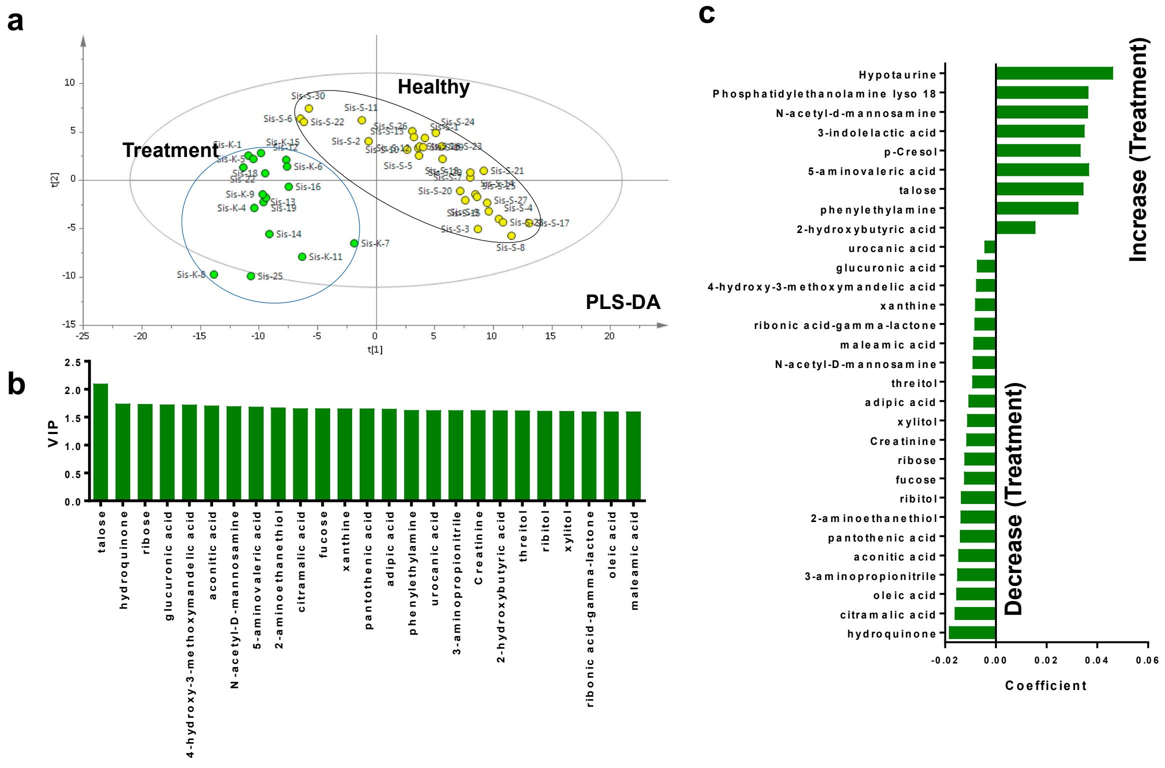

Figure S1.8. Multivariate analyses of data obtained from urine metabolomics analysis by GC-MS, LC-qTOF-MS and LC-MS/MS treatment group vs healthy group) **a)** PLS-DA score graph (R^2: 0.964, Q^2: 0.820) **b)** VIP graph showing most important metabolites leading differences in data analysis **c)** Coefficient graph showing increasing and decreasing metabolites between groups

When results of false classification analyses according to PLS-DA method were evaluated, it was observed that the patient versus healthy groups were differentiated from each other with a high sensitivity (>92%). It was even 100% between patients versus treatment groups and treatment versus healthy groups **(Table S1.6)**.

Table S1.6. False classification analyses for urine samples according to the PLS-DA analysis

	Members	Correct	Patient	Healthy
**Patient**	14	92.86%	13	1
**Healthy**	30	100%	0	30
Fishers prob.	$2.7x10^{-10}$			

	Members	Correct	Patient	Treatment
**Treatment**	17	100%	17	0
**Patient**	14	100%	0	14
Fishers prob.	$3.8x10^{-9}$			

	Members	Correct	Treatment	Healthy
**Treatment**	17	100%	17	0
**Healthy**	30	100%	0	30
Fishers prob.	$3.6x10^{-13}$			

S2. Data Analysis of LC-qTOF-MS based proteomics analysis results

Shotgun proteomics analyses with LC-qTOF-MS were performed from serum samples of patients (n=61) in those 12 patients with newly diagnosed cystinosis, 20 patients with already diagnosed and treatment and 29 healthy. 65 proteins that were common in the study groups were defined **(Table S1.7).**

Table S1.7. Statistical analysis results of common proteins found in the all groups

Proteins	Genes	T-test*		
		**Patient group vs treatment group**	**Patient group vs. healthy group**	**Treatment group vs. healthy group**
**Afamin**	*AFM*	0.162	0.813	0.165
**Alpha-1-acid glycoprotein 1**	*ORM1*	0.059	0.111	0.511
**Alpha-1-acid glycoprotein 2**	*ORM2*	0.198	0.675	0.280
**Alpha-1-antichymotrypsin**	*SERPINA3*	0.076	0.041	0.799
**Alpha-1-antitrypsin**	*SERPINA1*	0.771	0.009	0.061
**Alpha-1B-glycoprotein**	*A1BG*	0.853	0.117	0.268
**Alpha-2-antiplasmin**	*SERPINF2*	0.138	0.870	0.152
**Alpha-2-HS-glycoprotein**	*AHSG*	0.793	0.001	0.026
**Alpha-2-macroglobulin**	*A2M*	0.778	0.302	0.520
**Angiotensinogen**	*AGT*	0.357	0.009	0.414

Proteins	Genes	T-test*		
		Patient group vs treatment group	Patient group vs. healthy group	Treatment group vs. healthy group
**Antithrombin-III**	*SERPINC1*	0.506	0.089	0.434
**Apolipoprotein A-I**	*APOA1*	0.151	0.509	0.037
**Apolipoprotein A-II**	*APOA2*	0.024	0.003	0.758
**Apolipoprotein A-IV**	*APOA4*	0.432	0.002	0.001
**Apolipoprotein B-100**	*APOB*	0.333	0.304	0.078
**Apolipoprotein C-III**	*APOC3*	0.691	0.030	0.229
**Apolipoprotein E**	*APOE*	0.961	0.722	0.727
**Beta-2-glycoprotein 1**	*APOH*	0.646	0.006	0.065
**C4b-binding protein alpha chain**	*C4BPA*	0.583	0.703	0.410
**Carboxypeptidase N subunit 2**	*CPN2*	0.364	0.859	0.438
**Ceruloplasmin**	*CP*	0.237	0.441	0.050
**Clusterin**	*CLU*	0.895	0.396	0.560
**Complement C1r subcomponent**	*C1R*	0.717	0.041	0.020
**Complement C3**	*C3*	0.709	0.916	0.828
**Complement C4-B**	*C4B*	0.627	0.466	0.863
**Complement C5**	*C5*	0.351	0.132	0.733
**Complement component C6**	*C6*	0.733	0.365	0.660
**Complement component C9**	*C9*	0.090	0.554	0.282
**Complement factor B**	*CFB*	0.453	0.664	0.724
**Corticosteroid-binding globulin**	*SERPINA6*	0.897	0.820	0.753
**Fibronectin**	*FN1*	0.474	0.195	0.679
**Galectin-3-binding protein**	*LGALS3BP*	0.470	0.664	0.135
**Gelsolin**	*GSN*	0.670	0.513	0.220
**Haptoglobin**	*HP*	0.560	0.109	0.060
**Hemoglobin subunit beta**	*HBB*	0.365	0.017	0.221
**Hemopexin**	*HPX*	0.550	0.000	0.001
**Histidine-rich glycoprotein**	*HRG*	0.796	0.431	0.320
**Histone H2AX**	*H2AFX*	0.253	0.070	0.748
**Immunoglobulin heavy constant gamma 1**	*IGHG1*	0.198	0.330	0.087
**Immunoglobulin kappa constant**	*IGKC*	0.182	0.534	0.043
**Immunoglobulin lambda-like polypeptide 5**	*IGLL5*	0.513	0.080	0.037
**Inter-alpha-trypsin inhibitor heavy chain H1**	*ITIH1*	0.770	0.023	0.094
**Inter-alpha-trypsin inhibitor heavy chain H2**	*ITIH2*	0.330	0.030	0.007
**Inter-alpha-trypsin inhibitor heavy chain H3**	*ITIH3*	0.562	0.000	0.010
**Inter-alpha-trypsin inhibitor heavy chain H4**	*ITIH4*	0.498	0.000	0.004

Proteins	Genes	T-test*		
		Patient group vs treatment group	Patient group vs. healthy group	Treatment group vs. healthy group
**Kininogen-1**	*KNG1*	0.578	0.059	0.281
**Leucine-rich alpha-2-glycoprotein**	*LRG1*	0.053	0.042	0.981
**Lumican**	*LUM*	0.788	0.484	0.729
**Mediator of RNA polymerase II transcription subunit 12**	*MED12*	0.455	0.007	0.127
**N-acetylmuramoyl-L-alanine amidase**	*PGLYRP2*	0.818	0.448	0.639
**PHD finger protein 14**	*PHF14*	0.831	0.001	0.011
**Plasma protease C1 inhibitor**	*SERPING1*	0.080	0.025	0.576
**Plasminogen**	*PLG*	0.631	0.281	0.614
**Platelet basic protein**	*PPBP*	0.948	0.973	0.958
**Protein AMBP**	*AMBP*	0.567	0.000	0.000
**Prothrombin**	*F2*	0.441	0.171	0.031
**Retinol-binding protein 4**	*RBP4*	0.465	0.053	0.320
**Serotransferrin**	*TF*	0.696	0.008	0.059
**Serum amyloid P-component**	*APCS*	0.455	0.686	0.327
**Serum paraoxonase/arylesterase 1**	*PON1*	0.285	0.586	0.706
**Sex hormone-binding globulin**	*SHBG*	0.383	0.000	0.000
**Transthyretin**	*TTR*	0.776	0.111	0.280
**Vitamin D-binding protein**	*GC*	0.088	0.000	0.032
**Vitronectin**	*VTN*	0.908	0.429	0.557
**Zinc-alpha-2-glycoprotein**	*AZGP1*	0.071	0.002	0.540

*** p≤0.05 is statistically significant.**

Pathway analysis

Joint pathway analyzes based on the significantly altered metabolites and proteins (p <0.05) using the Metaboanalyst (ver. 5.0) platform. Pathways varying between patient groups and treatment and healthy groups were given in **Table S1.8 and 9**, respectively.

Table S1.8. Joint pathway analysis results between patients and treatment groups

Pathway Name	Match Status*	p value	-log(p)	FDR**
Central carbon metabolism in cancer	11/106	1.22×10^{-12}	11.913	4.04×10^{-10}
ABC transporters	11/183	4.8×10^{-10}	9.3163	7.99×10^{-8}
Citrate cycle (TCA cycle)	6/50	9.37×10^{-8}	7.0283	$1.03X 10^{-5}$

Glyoxylate and dicarboxylate metabolism	7/92	1.82 x 10^{-7}	6.7408	1.50X 10^{-5}
beta-Alanine metabolism	5/63	9.54 x 10^{-6}	5.0204	5.69X 10^{-4}
Alanine, aspartate and glutamate metabolism	5/64	1.03 x 10^{-5}	4.9866	5.69X 10^{-4}
Pyruvate metabolism	5/70	1.60 x 10^{-5}	4.7949	7.58X 10^{-4}
Phenylalanine metabolism	5/77	2.56 x 10^{-5}	4.5926	1.06X 10^{-3}
Glucagon signaling pathway	6/132	2.90 x 10^{5}	4.5374	1.07X 10^{-3}
Protein digestion and absorption	6/142	4.38 x 10^{5}	4.3586	1.45X 10^{-3}
Aminoacyl-tRNA biosynthesis	5/118	1.96 x 10^{-4}	3.7068	5.91X 10^{-3}
Valine, leucine and isoleucine biosynthesis	3/27	2.46 x 10^{-4}	3.6097	6.30X 10^{-3}
Tryptophan metabolism	5/124	2.48 x 10^{-4}	3.6062	6.30X 10^{-3}
Arginine and proline metabolism	5/128	2.87 x 10^{-4}	3.5422	6.78X 10^{-3}
Ascorbate and aldarate metabolism	4/76	3.94 x 10^{-4}	3.4042	8.70X 10-^{3}
Taurine and hypotaurine metabolism	3/33	4.49 x 10^{-4}	3.3474	9.30X 10^{-3}
Mineral absorption	4/87	6.59 x 10^{-4}	3.1811	1.28X 10^{-2}
Nicotinate and nicotinamide metabolism	4/92	8.14 x 10^{-4}	3.0896	1.50X 10^{-2}
Lysine degradation	4/115	1.86 x 10^{-3}	2.7298	3.25X 10^{-2}
Cholesterol metabolism	3/60	2.58 x 10^{-3}	2.5878	4.28X 10^{-2}
Primary bile acid biosynthesis	3/64	3.11 x 10^{-3}	2.5079	4.88X 10^{-2}
Pentose phosphate pathway	3/65	3.25 x 10^{-3}	2.4888	4.88X 10^{-2}

*Number of metabolites in the data set/ Number of metabolites in the pathway. ** False Discovery Rate

Table S1.9. Joint pathway analysis results between patients and healthy groups

Pathway Name	Match Status*	p value	-log(p)	FDR**
ABC transporters	29/183	2.2 x 10^{-26}	25.65	7.5 x 10^{-24}
Central carbon metabolism in cancer	18/106	3.8 x 0^{-17}	16.42	6.3 x 10^{-15}
Protein digestion and absorption	17/142	1.3 x 10^{-13}	12.90	1.4 x 10^{-11}
Mineral absorption	14/87	3.2 x 10^{-13}	12.49	2.7 x 10^{-11}
Aminoacyl-tRNA biosynthesis	15/118	1.6 x 10^{-12}	11.81	1.0 x 10^{-10}
Glycine, serine and threonine metabolism	11/90	2.6 x 10^{-9}	8.58	1.4 x 10^{-7}
Valine, leucine and isoleucine biosynthesis	7/27	9.8 x 10^{-9}	8.01	4.6 x 10^{-7}
Tyrosine metabolism	11/114	3.3 x 10^{-8}	7.49	1.4 x 10^{-6}
Phenylalanine metabolism	9/77	1.2 x 10^{-7}	6.94	4.3 x 10^{-6}
Alanine, aspartate and glutamate metabolism	8/64	3.6 x 10^{-7}	6.45	1.2 x 10^{-5}
Glyoxylate and dicarboxylate metabolism	9/92	5.5 x 10^{-7}	6.26	1.6 x 10^{-5}

Cysteine and methionine metabolism	9/112	2.9×10^{-6}	5.54	8.0×10^{-5}
Phenylalanine, tyrosine and tryptophan biosynthesis	6/40	3.7×10^{-6}	5.43	9.4×10^{-5}
Arginine biosynthesis	6/44	6.6×10^{-6}	5.18	1.6×10^{-4}
Taurine and hypotaurine metabolism	5/33	$2{,}4 \times 10^{-5}$	4.63	5.2×10^{-4}
Cholesterol metabolism	6/60	4.1×10^{-5}	4.39	8.4×10^{-4}
Arginine and proline metabolism	8/128	6.5×10^{-5}	4.19	0.0013
Sulfur metabolism	5/43	8.8×10^{-5}	4.05	0.0016
Tryptophan metabolism	7/124	3.5×10^{-4}	3.45	0.0062
Pentose phosphate pathway	5/65	6.3×10^{-4}	3.20	0.0104
Ascorbate and aldarate metabolism	5/76	0.0013	2.89	0.0202
Biosynthesis of unsaturated fatty acids	5/79	0.0015	2.82	0.0222
Pantothenate and CoA biosynthesis	4/47	0.0015	2.81	0.0222
Citrate cycle (TCA cycle)	4/50	0.0019	2.71	0.0268
Pentose and glucuronate interconversions	5/89	0.0026	2.59	0.0342
Nicotinate and nicotinamide metabolism	5/92	0.0030	2.53	0.0379
Glutathione metabolism	5/94	0.0033	2.49	0.0401

*Number of metabolites in the data set/ Number of metabolites in the pathway. ** False Discovery Rate
