# Supplementary material for: Metabolomic Analyses to Identify Candidate Biomarkers of Cystinosis
Source: Int J Mol Sci. 2023 Jan 30;24(3):2603. doi: 10.3390/ijms24032603 (PMC9916752; doi:10.3390/ijms24032603)
Supplement: Supplementary file 1 [file ijms-24-02603-s001.zip › Supplementary File S2- Methods.pdf]

S1. Material and Method

S1.1. Metabolomic and Proteomics Analysis

Proteomics analyses from serum samples were performed using LC-qTOF-MS, whereas metabolomic analyses from plasma and urine samples were performed using GC-MS, LC-qTOF-MS, and LC-MS/MS **(Figure S2.1.)**.

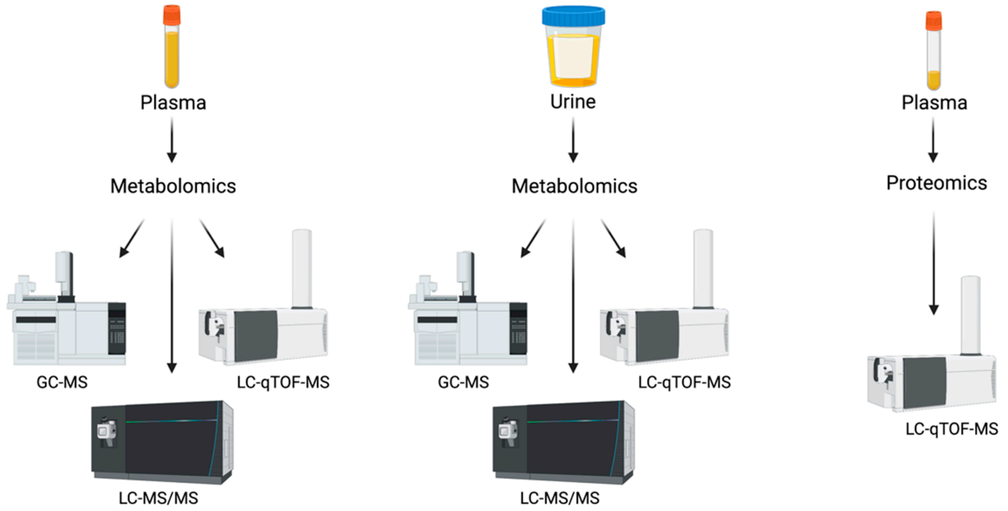

Figure. S2.1 Analytical techniques used for metabolomics and proteomics analysis based on the sampel type

S1.1.1. GC-MS based metabolomics analysis

GC-MS based metabolomics studies were performed as described previously [1, 2]. Briefly, samples stored at -80 °C were brought to room temperature and evaporated to dryness in a vacuum centrifuge. The dried samples were methoxylated by adding 20 µL of methoxyamine hydrochloride in pyridine (20 mg/mL) for 90 min at 30 °C. Then 80 µL of N-methyl-N-trimethylsilyl trifluoroacetamide + trimethylchlorosilane (MSTFA + 1% TMCS) was added to the samples at room temperature and was derivatized at 37 °C for 30 min. The derivatized samples were transferred to silylated GC-MS vials and then analyzed with a GC-MS (GC-MS QP-2010 Ultra, Shimadzu, Japan) system using DB5-MS (5% diphenyl/95 % dimethylpolysiloxan) column (30 m + 10 m DuraGuard × 0.25 mm i.d. and 0.25-µm film thickness) under the optimized conditions specified in **Table S2.1.** Representative chromatograms were given in the **Figure S2.2** for plasma and **Figure S2.3** for urine.

Table S2.1. GC-MS conditions for metabolomics analysis

**Column**	**DB5-MS column (30 m +10 m duraguard; 0.25 mm i.d. and 0.25 µm film thickness)**
**Oven Temperature Program**	60 °C (hold 1 min) to 325 °C (hold 10 min) at a rate of 10 °C min.
**Injection volume**	1 µL (Splitless)
**Carrier gas**	Helium 1 mL/min
**MSD transfer line temp.**	290 ºC
**Solvent delay time**	5.90 min
**Mass range**	50-650 dalton

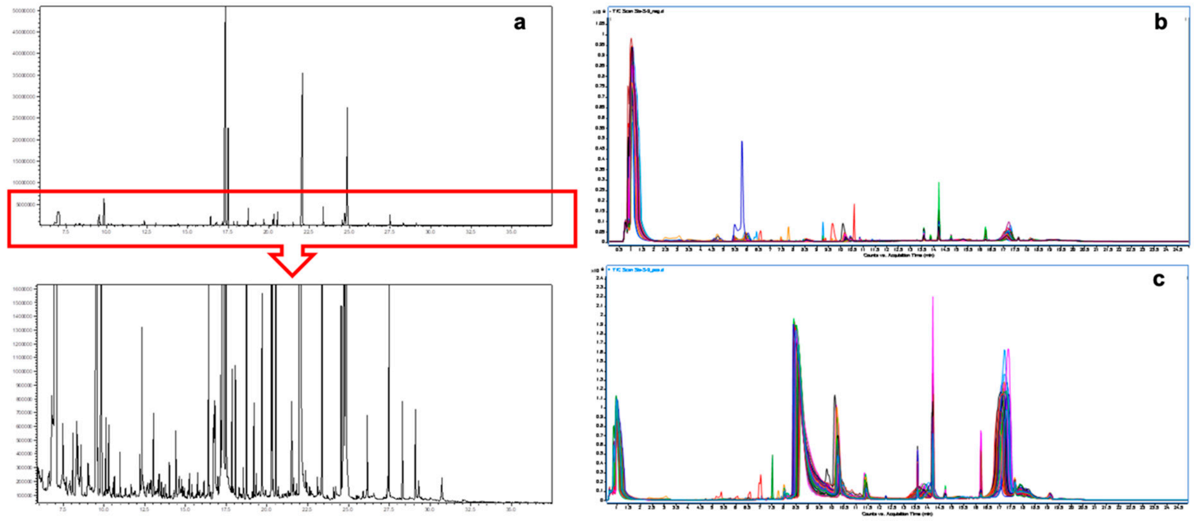

Figure S2.2. Representative chromatograms of plasma metabolomics analyses. a) GC-MS b) LC-qTOF-MS (negative ionization) c) LC-qTOF-MS (pozitive ionization)

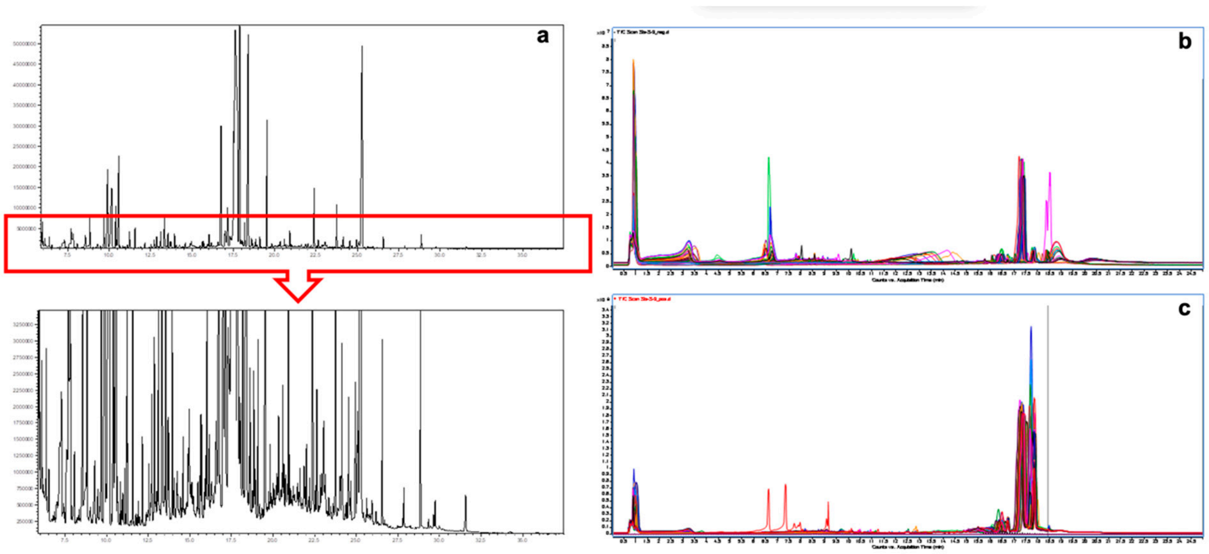

Figure S2.3. Representative chromatograms obtained from urine metabolomics analyses. a) GC-MS b) LC-qTOF-MS (negative ionization) c) LC-qTOF-MS (positive ionization)

.

S1.1.2. LC-qTOF-MS based metabolomics analysis:

LC-qTOF-MS-based metabolomics studies were carried out as described previously [1, 3]. Briefly, - After cooling to room temperature, the samples were evaporated to dryness in a vacuum centrifuge and re-dissolved in a water:acetonitrile (50:50, v/v) mixture containing 0.1% formic acid. The chromatographic separation of metabolites was performed by a C18 (100 x 2.1 mm, 2.7 µm) column. The mobile phase consisted of (A) water containing 0.1% FA and (B) acetonitrile containing 0.1% FA and the analyses were carried out using LC-qTOF-MS (Agilent 6530) at a flow rate of 0.3 mL/min with a gradient elution program in positive and negative modes. **(Table S2.2)**. The LC-qTOF-MS instrument parameters were given in **Table S2.3**. The selected QC samples by pooling of all samples were analyzed with different energies (10, 20, and 40 V) in targeted MS/MS mode to identify the peaks. Total ion chromatograms of LC-qTOF-MS based metabolomics analysis were given in the **Fig. S2.2b-c.** for plasma and **Fig. S2.3b-c.** for urine.

Table S2.2. Gradient elution program

Time (min)	% Mobile Phase B*
0	10
1	10
14	90
15	90
20	10
25	10

*% 0.1 FA in acetonitrile

Table S2.3. LC-qTOF-MS instrument parameters.

	Positive Ionization	Negative Ionization
**Mass range**	50-1700 amu	50-1700 amu
**Scan Speed (spectrum/s)**	2	2
**Spray voltage (kV)**	3500	3500
**Skimmer voltage (V)**	65	65
**Gas temperature (°C)**	325	325
**Gas flow rate (L/min)**	10	10
**Nebulizer (psig)**	45	45

S1.1.3. Proteomics analysis

ProteoPrep20 Plasma Immunodepletion Kit was used for optimal removal of the 12 highest abundance plasma proteins (albumin, IgG, α1-Acid glycoprotein, α1-antitrypsin, α2-macroglobulin, apolipoprotein A-I, apolipoprotein A-II, fibrinogen, haptoglobin, IgA, IgM, transferrin). The depletion protocols were performed according to the manufacturer's instructions. The depleted plasma was digested as described previously [2]. Briefly, protein pellets were redissolved in 100 mM ammonium bicarbonate containing 20% methanol, vortexed, and reduced for 15 minutes at 56 °C with 200 mM dithiothreitol (DTT). After that, samples were treated with 50 mM iyodoacetamide (IAA) for 30 minutes at room temperature in the dark and digested with trypsin (1:100, w/w) for one night at 37 °C. The chromatographic separations were carried out with C18 (150 mm x 0.5 mm, 5 µm) column. With a gradient mobile phase consisting of a mixture of 0.1% formic acid in water and 0.1% formic acid in acetonitrile at a flow rate of 0.07 mL/min. The mobile phase consisted of (A) water containing 0.1% FA and (B) acetonitrile containing 0.1% FA. The analyses were carried out using LC-qTOF-MS (Agilent 6530) at a flow rate of 0.07 mL/min with a gradient elution program **(Table S2.4)** in positive ionization mode using auto MS/MS. Tandem MS data of peptides with a threshold of 1000 counts and above were collected. Instrument parameters were presented in **Table S2.5**. The injection volume was 7 µL and the column temperature was maintained at 60 °C. The total run time was 170 min. Total ion chromatograms of LC-qTOF-MS based proteomics analysis was showed **Figure S2.4.**,

Table S2.4. Gradient elution program

Time (min)	% Mobile Phase B*
0	1
100	50
115	90
120	90
130	1
170	1

*% 0.1 FA in acetonitrile

Table S2.5. LC-qTOF-MS instrument parameters.

Mass Scale (MS)	200-1700 akb
**Mass Scale (MS/MS)**	50-1700 akb
**Scan Speed (spectrum/sn)**	3 (MS), 5 (MS/MS)
**Spray voltage (kV)**	3500
**Skimmer voltage (V)**	65
**Gas Temperature (°C)**	300
**Gas Flow Rate (L/dakika)**	8
**Nebulizer (psig)**	35
**Collision energy (eV)**	45
**Max Precursors Per Cycle**	10

**Charges**	+1, +2, +3, >+3

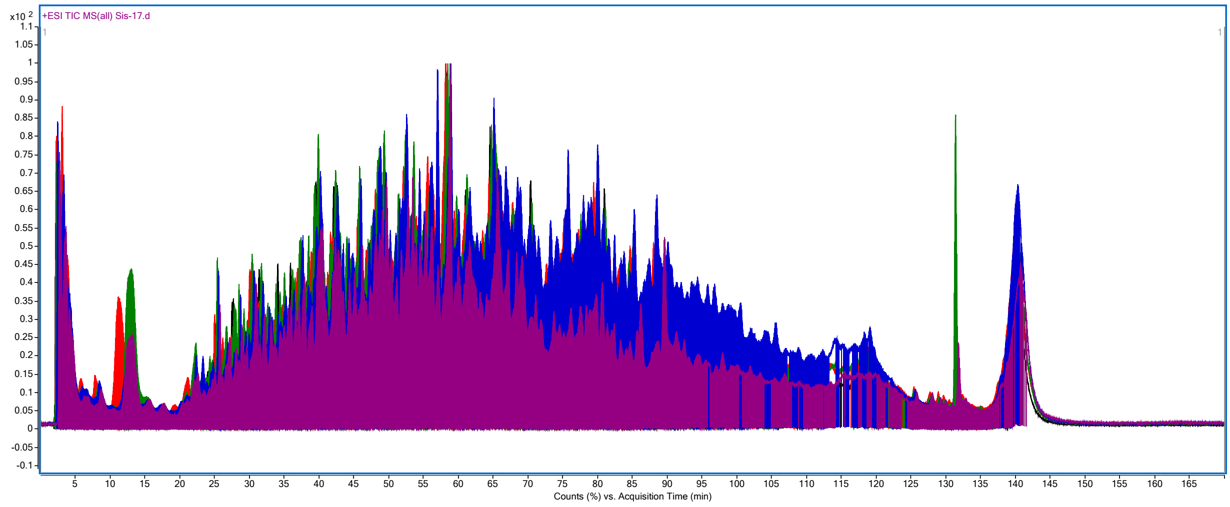

Figure S2.4. Representative chromatograms from plasma proteomics analysis

S1.1.4. Sulfur pathway analysis

The LC-MS/MS system consisted of a triple quadrupole mass spectrometer (Shimadzu 8030, Japan) equipped with an electrospray ionization source (ESI). The mass spectrometer was operated in the MRM at positive ionization mode **(Table S2.6)**. The gas flow rate and heat block temperature were 3 L/min and 400 °C, respectively. The separations were performed on MerckSeQuant ZIC-HILIC (100 x 4.6mm, 5 µm) with a mobile phase consisting of a mixture of (A) 0.1% formic acid in water and (B) 0.1% formic acid in acetonitrile at 0.35 mL/min flow rate in gradient elution mode**(Table S2.7).** The injection volume was 10 µL and the column temperature was maintained at 30°C.

Table S2.6. MRM paramters

Standard	*m/z*	Q1 (V)	CE	Q3 (V)
**Cystein**	121.80→ 59.10*	-13	-22	-11
	121.80→ 76.20	-14	-17	-30
**Cystin**	242.0→152.1*	-25	-15	-15
	242.0→74.0	-25	-29	-15
**Taurine**	126.10 → 107.9*	-16	-21	-18
	126.00 → 44.90	-12	-15	-30
**Hypotaurine**	110.00 → 65.10*	-12	-21	-16
	110.00 → 45.10	-12	-15	-30
**Serine**	106.0→60.1*	-15	-25	-15
	106.0→70.0	-12	-18	-15
**Glutathione**	307.90 → 179.10*	-18	-13	-12
	307.90 → 162.00	-11	-19	-17

**Cysteamine**	77.8→61.0*	-13	-15	-11
	77.8→35.2	-12	-25	-12
**Methionine**	149.8 →104.05*	-10	-15	-11
	149.8→56.05	-10	-21	-13
**Phenylalanine-1-^{13}C IS)**	167.1→121.1*	-15	-15	-24
	167.1→ 104	-10	-27	-22
**Cystin -1,1′-$^{13}C_2$ (IS)**	243.1→152.7*	-18	-19	-25
	243.1→120.9	-12	-14	-30
**Cystein -1-^{13}C (IS)**	123→59.1*	-13	-22	-11
	123→76.2	-14	-17	-30

* Quantifacition, CE: Collision energy, Q1 (V): First quadrupole voltage, Q3 (V): Third quadrupole voltage, IS: Internal standard

Table S2.7. Gradient elution program

Time (min)	% Mobile Phase B*
1	95
5	5
9	5
10	95
12	95

*% 0.1 FA in acetonitrile

[1] Nemutlu, E.; Orgul, G.; Recber, T.; Aydin, E.; Ozkan, E.; Turgal, M.; Alikasifoglu, M.; Kir, S.; Beksac, M. S. Metabolic Infrastructure of Pregnant Women With Trisomy 21 Fetuses; Metabolomic Analysis. *Z Geburtshilfe Neonatol*, **2019,** *223* (5), 297-303.
[2] Eylem, C. C.; Yilmaz, M.; Derkus, B.; Nemutlu, E.; Camci, C. B.; Yilmaz, E.; Turkoglu, M. A.; Aytac, B.; Ozyurt, N.; Emregul, E. Untargeted multi-omic analysis of colorectal cancer-specific exosomes reveals joint pathways of colorectal cancer in both clinical samples and cell culture. *Cancer Lett*, **2020,** *469*, 186-194.
[3] Nemutlu, E.; Zhang, S.; Xu, Y. Z.; Terzic, A.; Zhong, L.; Dzeja, P. D.; Cha, Y. M. Cardiac resynchronization therapy induces adaptive metabolic transitions in the metabolomic profile of heart failure. *J Card Fail*, **2015,** *21* (6), 460-9.
